# Recent Advances in the Fabrication of Membranes Containing “Ion Pairs” for Nanofiltration Processes

**DOI:** 10.3390/polym9120715

**Published:** 2017-12-14

**Authors:** Yan-Li Ji, Bing-Xin Gu, Quan-Fu An, Cong-Jie Gao

**Affiliations:** 1 Center for Membrane and Water Science & Technology, Ocean College, Zhejiang University of Technology, Hangzhou 310014, China; yanliji@zjut.edu.cn (Y.-L.J.); 17857685176@163.com (B.-X.G.); gaocj@zjut.edu.cn (C.-J.G.); 2 Beijing Key Laboratory for Green Catalysis and Separation, College of Environmental and Energy Engineering, Beijing University of Technology, Beijing 100124, China

**Keywords:** nanofiltration, ion pairs, polyelectrolyte membranes, zwitterionic membranes, charged mosaic membranes

## Abstract

In the face of serious environmental pollution and water scarcity problems, the membrane separation technique, especially high efficiency, low energy consumption, and environmental friendly nanofiltration, has been quickly developed. Separation membranes with high permeability, good selectivity, and strong antifouling properties are critical for water treatment and green chemical processing. In recent years, researchers have paid more and more attention to the development of high performance nanofiltration membranes containing “ion pairs”. In this review, the effects of “ion pairs” characteristics, such as the super-hydrophilicity, controllable charge character, and antifouling property, on nanofiltration performances are discussed. A systematic survey was carried out on the various approaches and multiple regulation factors in the fabrication of polyelectrolyte complex membranes, zwitterionic membranes, and charged mosaic membranes, respectively. The mass transport behavior and antifouling mechanism of the membranes with “ion pairs” are also discussed. Finally, we present a brief perspective on the future development of advanced nanofiltration membranes with “ion pairs”.

## 1. Introduction

Nowadays, the shortage of fresh water and available energy has become an important issue restricting the development of human society [1,2]. Compared with conventional separation methods like distillation and extraction, membrane separation technology with the advantages of low energy consumption, environment protective properties, high efficiency, and easy operation has been widely applied in water/wastewater treatment, bio-/petrochemicals purification and separation, and the recycling of resource materials, etc. [3,4]. With the development of membrane technology, the market for membrane application is growing at a fast rate of 9% and the over market size is expected to be $26 billion by 2017 [5]. Nanofiltration is a pressure-driven membrane separation technology; the membrane pore size (<1.0 nm) and operation pressure (0.1–1.5 MPa) are in the intermediate range between reverse osmosis and ultrafiltration membranes. According to the separation mechanism, e.g., the sieving (steric hindrance) effect and the Donnan (electrostatic) effect, nanofiltration membranes are suitable for separating different valent salts and organic molecules (200~1000 Da). Nanofiltration membranes are usually fabricated into asymmetric or composite structures, and the selective layer of commercial membranes is mainly derived from cellulose acetate and polyamide [6,7]. Although some success has been achieved in the development of membranes with high flux and good selectivity, the energy consumption of nanofiltration is still much higher than the thermos-dynamic limit. In addition, membrane fouling can lead to a serious decline of permselectivity, ultimately increasing the energy demand, requiring additional costs for cleaning and maintenance, and shortening membrane lifetime [8]. Moreover, the stability and separation performance should be significantly improved for organic solvent nanofiltration (OSN), sometimes also referred to as solvent-resistant nanofiltration (SRNF) [9]. Thus, there is an important requirement to develop antifouling and stable nanofiltration membranes with supreme permeability and selectivity to meet the complicated requirements of actual applications.

**Figure 1:**
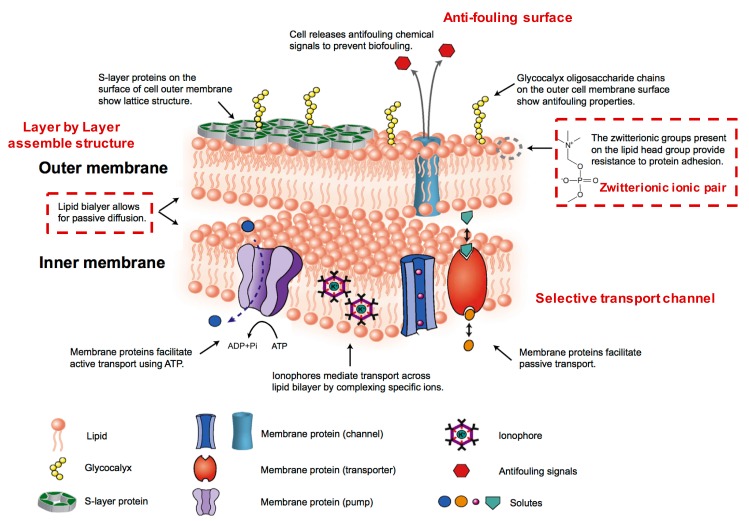
Biological membrane separation and antifouling strategies for an example of a gram-negative bacterial organism. Reproduced with permission from Reference [12].

‘Learn from nature’ has become a popular philosophy in the design and fabrication of separation membranes [10]. Among all the natural prototypes, cell membranes are always the most interesting and important due to their exquisite structure, excellent performance in mass transfer and energy transformation, as well as their supreme resistance to various foulants [11]. The cell membrane is mainly constructed by amphipathic phospholipid molecules to form a lipid bilayer structure, which is a typical example of molecular self-assembly in nature (as shown in Figure 1). The layer-by-layer self-assembly method as an intelligent and bioinspired strategy has been explored to fabricate separation membranes with multiple components with a nanometer-scale thickness [12]. Water, ions, and small organic molecules can be transported quickly through cell membranes with exceptional selectivity. There are many types of water channels, for example, aquaporin 1 (AQP1) water channels allow water to move freely and permeate much faster (3 × 10^9^ water molecules per subunit per second) across the cell membrane, but not other small organic molecules, ions, or even protons [13,14]. Additionally, zwitterionic phosphatidylcholine with “ion pairs”, as the major phospholipid located on the outer layer of cell membranes, displays excellent biocompatibility and antifouling properties. As such, the self-assembly of cell membranes provides special and exquisite examples for the design and fabrication of separation membranes with outstanding performance.

Polyelectrolytes are polymers with charged monomer groups that can classified into positively charged polyelectrolytes, negatively charged polyelectrolytes, and zwitterionic polyelectrolytes [15]. The oppositely charged polyelectrolytes can be used to prepare thin film by electrostatic layer-by-layer (LBL) assembly technology. Similar to the construction of the cell membrane, the LBL films can be fabricated in nanometers on a support with controlled thickness. The charged character and ionic cross-linking density can be easily tuned with different types of polyelectrolytes and assembly conditions [16], resulting in an optimum separation performance to meet application requirements. Moreover, the cross-linking structure of ion pairs within LBL films is stable in most organic solvents, which has great potential in the area of organic solvent nanofiltration separation. With the control of ionic complex degree of oppositely charged polyelectrolytes, water-dispersible polyelectrolyte–polyelectrolyte complexes (PECs) were obtained [17]. Homogenous PECs (HPECs) can be used directly to prepare membranes by surface coating and cross-linking methods. The key issue for preparing HPEC nanofiltration membranes is to strike a balance between the charge density and hydrophilicity, which determines the membranes’ permeability and selectivity. In addition, HPECs are alternative candidates for preparing nanocomposite nanofiltration membranes; the nanomaterials could be well-dispersed in the HPEC casting solution, and there is good compatibility between the nanomaterials and the HPEC matrix, leading to an excellent separation performance. 

Bio-inspired by zwitterionic phosphatidylcholine head-groups existing in the phospholipid bilayer of cell membranes, zwitterionic polymers have become alternative candidates for preparing antifouling nanofiltration membranes. Zwitterionic polymers simultaneously containing both anionic and cationic groups can be classified into polybetaines and polyampholytes [18]. Polyampholytes have oppositely charged groups are located on different monomer units, while polybetaines have cationic/anionic groups on the same monomer units, e.g., poly(2-methacryloyloxyethyl phosphorylcholine) (PMPC), poly(sulfobetaine methacrylate) (PSBMA), and poly(carboxybetaine methacrylate) (PCBMA). Zwitterionic materials containing ion pairs have superior bio-compatibility and antifouling properties because they can adsorb an abundance water molecules via electrostatic interactions, and the denser and tighter hydration shell protects the membrane surface from contamination with foulants [19,20]. It has been suggested that the non-/antifouling materials should have high hydrophilicity, electric neutrality, and hydrogen bond acceptors which can bond high amounts of water molecules [21]. The strong hydrophilicity, uniformity of charge distribution, and charge neutrality are key to controlling the antifouling properties of zwitterionic materials [22]. Moreover, zwitterionic materials have responsive properties; the separation performance of their nanofiltration membranes can be tuned with varying operation conditions. In recent years, there has been research reporting that the separation performance and antifouling properties were improved via incorporating zwitterionic ion pairs into membranes [20,22]. 

Charged mosaic membranes are well known as membranes containing oppositely charged groups within the matrix [23]. The array of ion pairs induces the concurrent migrations of cations and anions along the respective fixed charges of membrane. Since 1932, the concept of the charge mosaic membrane was firstly proposed by Sollner, and many attempts have been made to develop such membranes from polymer blends, through polyelectrolytes interfacial polymerization, self-assembled block polymers, and LBL deposition/electrospun polymers [24,25]. Charged mosaic membranes containing equivalent amount of cationic and anionic groups, which would be likely to facilitate electrolyte transport through the membrane. As a result, the permeation of salts is enhanced by maintaining a high rejection to low molecular weight organics. This unique feature of the mosaic membrane is very fascinating in the application of separating salts and organic molecules.

**Figure 2:**
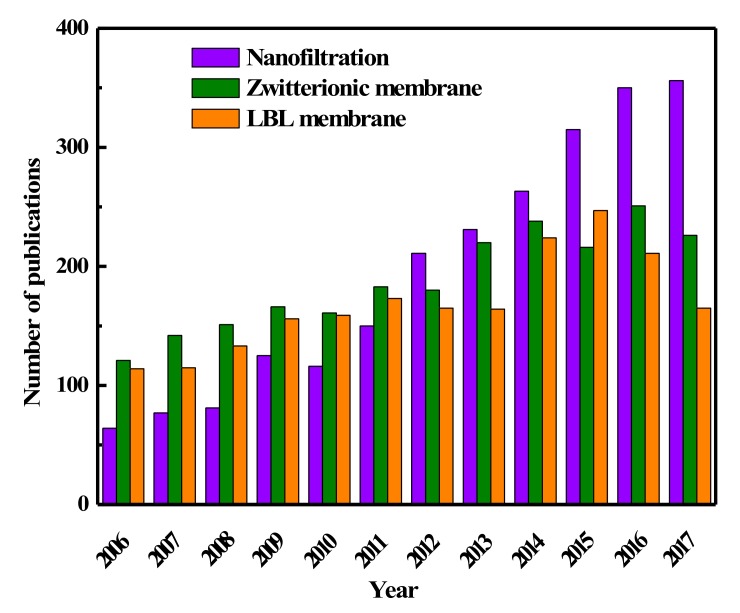
Record of the number of papers published in indexed journals between 2006 and 2015 containing the keywords “nanofiltration’’ or “zwitterionic membrane” or “layer by layer (LBL) membrane” in the title, abstract, or keywords. (Source: Web of Science, searched on 8 November 2017).

**Figure 3:**
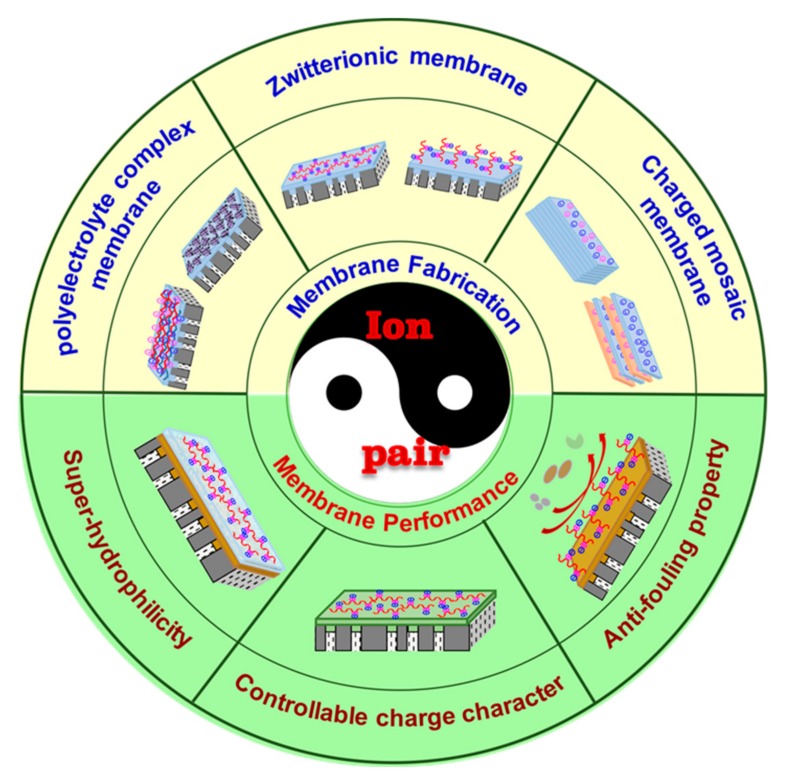
Overview of fabrication membranes containing ion pairs with improved permeability, selectivity, and antifouling properties.

Materials containing “ion pairs” have been widely used in new nanofiltration membranes exploration and traditional membranes modifications accompanied with many unique advantages. Research publications on nanofiltration membranes, zwitterionic membranes, and LBL membranes are illustrated in Figure 2, which confirms that increasing attention has been paid to the development of nanofiltration membranes and membranes containing “ion pairs”. This review first presents a brief introduction of the membranes containing “ion pairs”. Then, the effects of “ion pairs” characteristics on membrane performances including solvent permeability, solute selectivity, and antifouling property are discussed. After that we also review and critically discuss the various methods and the different factors affecting the fabrication of polyelectrolyte complex membranes, zwitterionic membranes, and charged mosaic membranes, respectively. The review includes the preparation and surface modification of nanofiltration membranes by LBL deposition, surface coating, interfacial polymerization, surface grafting, biomimetic adsorption, and so on. A schematic overview of all these unique features of “ion pairs” and the strategies for membrane construction is presented in Figure 3. Finally, a brief perspective on the future development of advanced nanofiltration membranes with “ion pairs” is presented.

## 2. Influence of “Ion Pairs” Characteristic of Nanofiltration Performance

### 2.1. Super-Hydrophilicity of “Ion Pairs” Affects Membrane Permeation

**Figure 4:**
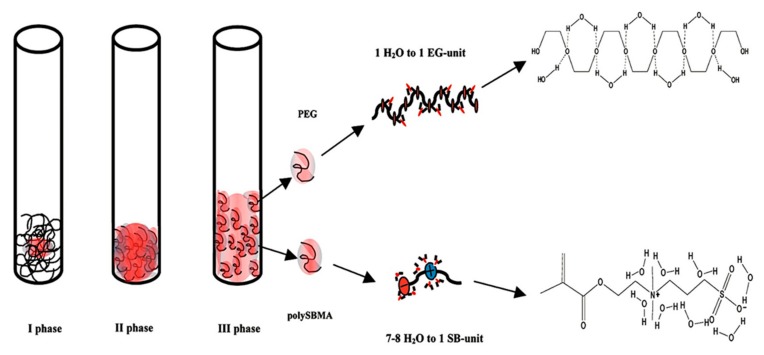
A proposed model of the hydration process of both PEG and polySBMA polymers. The red dots with two black sticks represent one water molecule, while the black lines represent linear polymer chains. The hydration of polymers might go through three stages with increasing water content: water molecules tightly bind to polymers, saturate binding sites of polymers, and finally swell and dissolve polymers. One water molecule could bind with one ethylene glycol (EG) unit through two H bonds, while about eight water molecules could fully hydrate an sulfobetaine (SB) group through electrostatic force. Reproduced with permission from Reference [29].

The permeation of nanofiltration membranes is primarily influenced by the hydrophilicity/hydrophobicity of membrane materials. For example, the combination of the hydrophilicity of membranes and the solvent polarity determines the permeation of aqueous/solvents through hydrophilic/hydrophobic nanofiltration membranes [26,27]. It has been demonstrated that the permeate of aqueous or polar solvents in nanofiltration membranes containing “ion pairs” was much higher than that of non-polar solvents. Zwitterionic polymers and poly(ethylene glycol) (PEG) are two main classes of hydrophilic materials that have been widely used for modifying separation membranes to enhance their hydrophilicity [28]. In order to know the interaction between water molecules and membranes containing zwitterionic ion pairs, hydrated water within polymers has been studied in depth [29,30]. Poly(sulfobetaine methacrylate) (polySBMA), a typical zwitterionic material, shows super-high hydrophilicity due to the strong interaction between ion pairs and water molecules. Compared with PEG, water molecules interacting with the SB unit are more tightly adhered than those on the ethylene glycol (EG) unit before saturation [29]. In addition, there are more water molecules adsorbed on polySBMA chains than those on PEG, e.g., the EG unit includes an oxygen atom that could integrate with one water molecule via hydrogen bonding interactions, while the SB unit consists of a positively charged group and a negatively charged group integrated with at most eight water molecules via electrostatic interactions (Figure 4). Moreover, the dipole array of the additional water molecules in the hydration shell formed with SB units are closer to free water, which makes the water molecules easily transported through zwitterionic membranes. 

The hydrophilicity and water permeability of membranes could be enhanced by incorporating zwitterionic ion pairs into membranes. Zhao et al. [31] grafted a zwitterionic polymer, poly(sulfobetaine methacrylate) (poly(SBMA)), onto a hydrophobic polypropylene membrane surface. The water contact angle was sharply decreased from 106° to 17.4° with an increasing atom transfer radical polymerization (ATRP) graft time for SBMA. The enhanced hydrophilicity is mainly due to the hydrophilic zwitterionic groups which can bond large amounts of water through electrostatic interactions. Wang et al. [32] modified aromatic polyamide membranes with poly(zwitterionic carboxybetaine methacrylate) (PCBMA); the water flux of the modified membrane increased 22.55% compared to that of the pristine membrane, while the salt rejection changed slightly. However, with further increasing the grafting degree of PCBMA, the water flux decreased a little. The flux first increases and then decreases with the increase of zwitterionic grafting degree, which can be explained by the improvement of hydrophilicity. Meanwhile, the hydrogen bonds of the aromatic polyamide chains were weakened and thus increased the water passage. On the other hand, the grafting of PCBMA would provide an increased resistance to mass transfer across the membrane. Thus, the grafting of zwitterionic polymers should be controlled with an optimum degree, resulting in a highest permeation flux and good rejection. An et al. [33,34] incorporated zwitterionic monomer (*N*-aminoethyl piperazine propane sulfonate, AEPPS) into polyamide membranes, and both the hydrophilicity and surface roughness of the membrane was enhanced. The zwitterionic AEPPS composed of ion pairs are capable of binding high amounts of free water; in addition, a greater membrane surface would make direct contact with water molecules and facilitate an increase of the water flux.

Thin films constructed with oppositely charged polyelectrolytes are also considered to be good candidates for aqueous/polar organic solvent nanofiltration [35], since the “ion pairs” structure within the thin film layer can provide high permeability for water and polar solvents. Moreover, the ionic cross-linking degree can be easily tuned with different types of “ion pairs” and fabrication conditions, thus there would be good separation performance for salts/charged molecules in polar solvents. Malaisamy et al. [36] fabricated ultrathin polyelectrolyte LBL film with 2.5 bilayers of poly(styrenesulfonate) (PSS)/protonated poly(allylamine) (PAH) or 3.5 bilayers of PSS/poly(diallyldimethylammonium chloride) (PDDA) on ultrafiltration substrates. The water permeability of LBL membranes can be easily tuned by adjusting the electrostatic interaction between oppositely charged polyelectrolytes. For example, PDDA has a significantly lower charge density compared to PAH, thus PSS/PDDA films contain a lower ionic cross-linking density than PSS/PAH coatings. The films composed of 3.5 and 4.5 PSS/PDDA bilayers exhibit a solution flux of 1.6–1.8 m^3^/(m^2^ day) along with sulfate rejections of 95–96% and a chloride/sulfate selectivity of 27–32. Li et al. [37] prepared polyelectrolyte LBL membranes with PDDA and sulfonated poly(ether ether ketone) (SPEEK), and the resulting membranes were used for the separation of charged aromatic dyes in aprotic solvents. There is a high chemical stability and high separation performances in aprotic solvents like tetrahydrofuran (THF), *N*,*N*-Dimethylformamide (DMF), and isopropanol (IPA). The cross-linking interaction between ion pairs makes the LBL membrane stable in solvents, and the different physical properties of solvents and the interaction between the membrane and solvent lead to a different solvent flux. 

### 2.2. Controllable Charge Character of “Ion Pairs” Affects Membrane Separation

**Figure 5:**
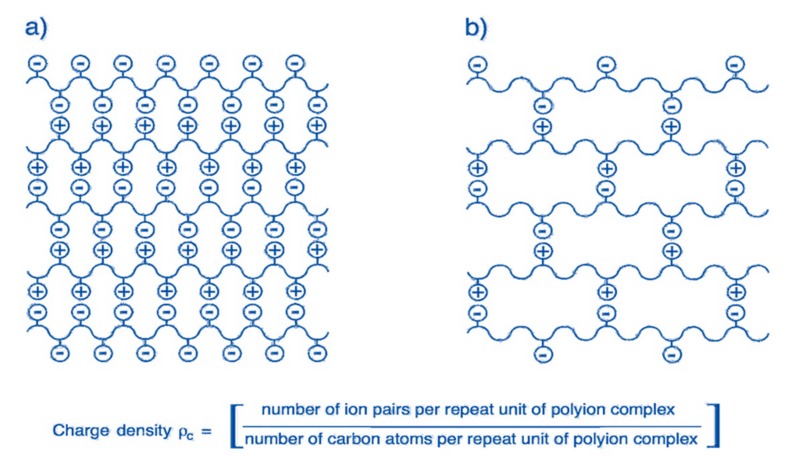
Simplified structure model of multilayered polyelectrolyte membrane of high (**a**) and low charge densities (**b**). The nanopores become larger and less hydrophilic as the charge density decreases. Possible chain interdigitation and incomplete neutralization are not considered. Reproduced with permission from Reference [41].

Nanofiltration membranes are usually negatively/positively charged and have pores with a size in the range of 0.3~1.0 nm. Thus, they combine both size and Donnan exclusion effects to control the solutes transport behaviors [6]. Due to the existence of intrinsic pores in nanofiltration membranes, the steric exclusion accounts for separating solutes with different sizes. Meanwhile, in the case of electrolyte solutions, charge effects play an important role in determining the separation performances of charged nanofiltration membranes when the size of solutes is much smaller than the pores of membranes [4]. The rejection performance is remarkably affected by the charged character of membranes and types of ionic species, in addition to the operation conditions such as the pH and ionic strength of feed solutions. Furthermore, because the dielectric constant of an aqueous solution is much higher than those in the membrane materials, there is an image force when the electrolytes enter the pores of membranes, thus the opposite interaction leads to an additional rejection for charged species. Polyelectrolyte multilayer films contain both cationic and anionic charges, the cross-linking between ion pairs makes a structural network of membranes [38,39], which are suitable for the separation of different valent salts and organic molecules. Jin et al. [40] investigated the separation performance of polyelectrolyte multilayer membranes prepared with oppositely charged polyvinylamine (PVA) and polyvinyl sulfate (PVS). It was found that the salt rejection increases in the series NaCl < Na_2_SO_4_ < MgCl_2_ < MgSO_4_, because there is a stronger electrostatic interaction between divalent ions and the membrane-bound charged groups in comparison to the monovalent groups. Interestingly, the MgCl_2_ rejection is higher than Na_2_SO_4_ rejection; the authors thought that this is because the magnesium ions have stronger electrostatic interaction with the sulfate ions of PVS than the permeating sulfate ions do with the ammonium ions of PVA. The separation performance is dominated by the electrostatic interaction between salts and ion pairs of polyelectrolyte multilayer membranes. Tieke et al. [41] investigated the relationship between the ionic cross-linked structure of polyelectrolyte multilayer membranes and their separation performance. The network structure is schematically shown in Figure 5, in which that the ion pairs represent the cross-linking sites and the pore size of the network can be tailored by varying the charge density of polyelectrolytes. For example, using polyelectrolytes of a high charge density such as PVA and PVS, a network with small pores is formed, while with PDDA and PSS, a network with larger pores is obtained. For PAH and PSS, a network with medium pore size can be expected. The mean pore size of polyelectrolyte multilayer films such as PDDA/PSS, PAH/PSS, and PVA/PVS was determined to be about 0.82, 0.67, and 0.54 nm, respectively. This indicates that the pore size of the membranes can be precisely adjusted with the choice of polyelectrolytes of different charge density. Thus, it is possible to tailor optimum conditions for efficient separation. Hong et al. [42] fabricated PSS/PAH multilayer films to separate neutral molecules and salts. The obtained membranes exhibit NaCl/sucrose selectivity of ~130, NaCl/dye selectivity >2200, and NaCl/glutamine selectivity of ~3.7. This is because the pore size produced by the ionic cross-linking of oppositely charged polyelectrolytes is larger than the molecular size of glutamine, thus the rejection decreases in the order of dyes > sucrose > glutamine. This work demonstrates the potential of such polyelectrolyte multilayer membranes in the selective separation of organic molecules and salts. 

**Figure 6:**
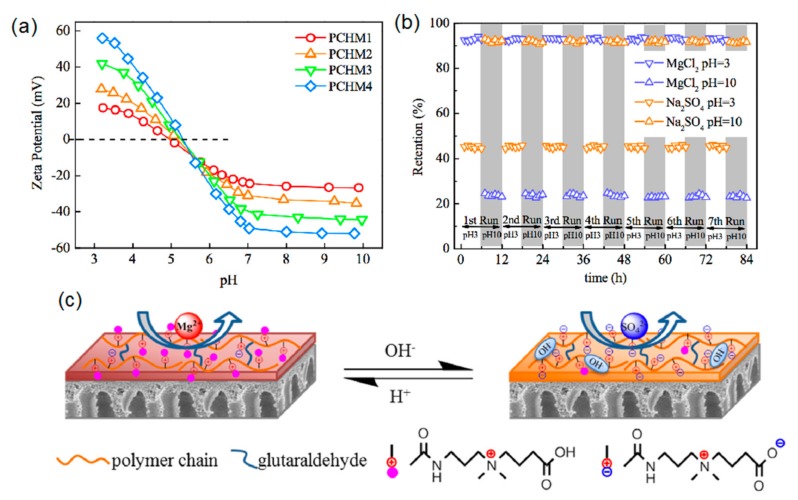
(**a**) Zeta potential varies with the pH of poly(carboxybetaine methacrylamide-*co*-*N*-(hydroxymethyl) acrylamide) membranes (PCHMs) tested with 1.0 mmol L^−1^ KCl aqueous solution at 25 °C; (**b**) Reversible change of retention of PCHM3 tested with 1 g L^−1^ MgCl_2_ and Na_2_SO_4_ under feed pH value oscillating between 3.0 (∇) and 10.0 (Δ) at 25 °C and 0.6 MPa (the washing time was not counted in the plot); (**c**) The schematic model for pH responsibility of PCHMs. Reproduced with permission from Reference [47].

Zwitterionic polyelectrolytes and weak acid/base polyelectrolytes have stimuli-responsive properties; their nanofiltration membranes’ pore size and surface properties can be tuned by the manipulation of the environmental and operational conditions, such as pH, temperature, ionic strength, light of the environment, etc. [43,44]. Zhao et al. [45] investigated the electrolyte-responsive behaviors of a zwitterionic sulfobetaine regenerated cellulose (RC) membrane, which was prepared by grafting poly(sulfobetaine methacrylate, PSBMA) on an RC membrane. Because the electrostatic attraction among ion pairs could be changed with the addition of inorganic salts, the intra-/inter-chain associations of zwitterionic PSBMA chains in RC membrane were varied, leading to a tunable permeation selectivity. This result showed that zwitterionic membranes could separate organic molecules with different sizes with the alteration of the electrolyte concentration in solution. Zwitterionic carboxybetaine polymers containing both quaternary ammonium groups and carboxylate groups are pH-responsive polymers. In deionized water or alkaline aqueous solutions, there are ion pairs structures in the polymers, while at low pH, the zwitterionic polymers transform into polycations because of the protonation of carboxylate groups [46]. An et al. [47] fabricated a novel type of pH-responsive nanofiltration membranes with poly(carboxybetaine methacrylamide-*co*-*N*-(Hydroxymethyl) acrylamide) (PCHs). As shown in Figure 6, there is a significant pH-responsive separation performance; the retention of PCH membranes (PCHMs) to Na_2_SO_4_ increases and the retention to MgCl_2_ decreases with increasing the feed pH value from 3.0 to 10.0. It is found that the pH-responsive behavior for PCHMs is mainly determined by the membrane surface charge. PCHMs have more negative charges at a higher feed pH value, resulting in a stronger electrostatic repulsion between SO_4_^2−^ ions and membranes, thus the Na_2_SO_4_ rejection of PCHMs becomes higher. In reverse, there is a lower MgCl_2_ rejection due to the electrostatic attraction between Mg^2+^ and negatively charged PCHMs. In addition, the separation performance of LBL membranes containing weak acid or weak base ion pairs can also be tuned by adjusting the pH value [48]. Dai et al. [49] used the partial Fischer esterification of poly(acrylic acid) to tailor the hydrophobicity and charge density of multilayer films containing PAH and derivatized poly(acrylic acid) (d-PAA). The separation performance of PAH/d-PAA films can be controlled by adjusting the hydrophobicity and charge density. The hydrophobicity of these films was increased with the increasing content of hydrophobic ester groups, which decreases their permeability to electrochemically active probe molecules. After hydrolysis, the density of –COO^−^ groups increases, which makes the membranes more permeable to positively charged Ru(NH_3_)_6_^3+^ than to Fe(CN)_6_^3−^. From previous research, it is found that the formation of an ion pairs structure of polyelectrolytes has a broad processing window, and can be easily tuned by adjusting environmental conditions to obtain the desired structure and separation performance for nanofiltration membranes.

The charged character of ion pairs within polyelectrolyte LBL membranes/charged mosaic membranes influences the ion-exchange capacity and permselectivity. Adusumilli et al. [50] investigated the charge character, ion-exchange, and transport selectivity of (PSS/PDDA)_n_ films. It is found that the ζ potentials of PSS/PDDA films terminate with the change of PSS from negative to positive with an increasing the number of adsorbed bilayers, probably because there are many anion-exchange sites inside the films. These changes in film properties dramatically affect ion transport through the films, where the Cl^−^/SO_4_^2−^ selectivity is >30 with (PSS/PDDA)_4_PSS films but only 3 with (PSS/PDDA)_6_PSS films. Thus, the ion-exchange capacity and salt selectivity of the multilayer membranes can be tuned by varying the ionic cross-linking density and the number of layers. Sollner proposed a phenomenological theory for ionic transport through charged mosaic membranes [51]; they reported that there are circulating currents between individual ion-exchange regions, thus the membrane shows negative osmosis and greater charged substances transport in comparison to the neutral molecules. Yamauchi et al. [52] studied the transport phenomenon of amino acids and sucrose through charged mosaic membranes on the basis of KCl transport. The results showed that the order of transport was KCl, LiCl > GluNa > Arg, Ala > glucose, sucrose. Moreover, the amino acid transport depends on both the charged character and molecular size; however, the non-electrolyte was rejected and sucrose permeated under all experimental conditions. Higa et al. [53] fabricated charged mosaic membranes from laminated structures of negatively/positively charged poly(vinyl alcohol) membranes. The authors found that the flux of salts and the permselectivity of membranes could be controlled by adjusting the charge density (numbers of ion pairs, a decrease in the domain size between the oppositely charged layers) and the cross-linking density (the annealing conditions). A novel type of inorganic–organic hybrid zwitterionic polymer was prepared by reacting 3-glycidoxypropyltrimethoxysilane with *N*-[3-(trimethoxysilyl) propyl] ethylene diamine, and subsequently with γ-butyrolactone [54]. The membranes containing both negatively and positively charged groups are used for desalting KCl solutions (0.0001–0.1 M) at an applied pressure of only 30 kPa, and the salt accumulation was 12–38% depending on the concentration, which ranged from 7.6 to 7550 mg/L. These results indicate that charged mosaic membranes with ion pairs are promising for the removal of salts and the separation of electrolytes and nonelectrolytes.

### 2.3. Fouling Resistance of “Ion Pairs” Affects Membrane Antifouling Performance

**Figure 7:**
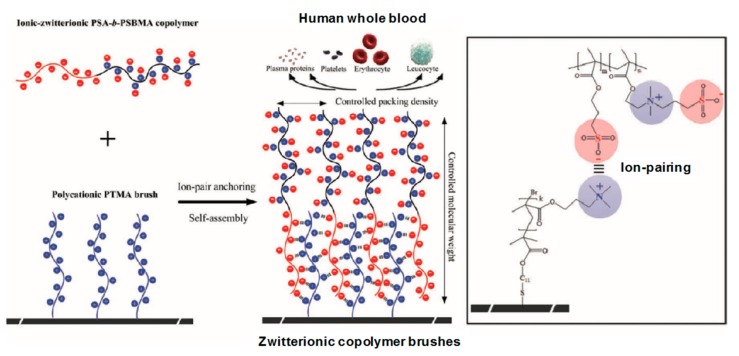
Ion-pair anchoring of zwitterionic copolymer brushes on the membrane surface. Reproduced with permission from Reference [63].

In the study of the cell membrane, it is found that the phospholipid bilayer has a strong resistance to the adsorption of exogenous proteins, polysaccharides, peptides, and other foulants. This excellent antifouling performance is mainly attributed to the hydrophilic property of zwitterionic head groups on the membrane surface, and the steric hindrance of nonpolar hydrophobic glycoproteins tails in the interior [55,56]. Inspired by the cell membrane, zwitterionic antifouling polymers containing the pendant groups of phosphobetaine, sulfobetaine, and carboxybetaine have been synthesized and used for preparing antifouling membranes [57,58]. In general, it is acknowledged that zwitterionic ion pairs on the membrane surface can adsorb a large amount of water molecules to form a “free-water” hydration shell via electrostatic interactions, which is an effective barrier to prevent the surface from directly contacting the foulants. Liu et al. [59] grafted a sulfobetaine polymer brush on polyamide thin-film membrane, and the zwitterionic polyamide membrane exhibited a lower surface roughness, higher hydrophilicity, and decreased surface charge. Chemical force microscopy showed that the foulant-membrane interaction force of the zwitterionic polyamide membrane is one order of magnitude smaller than that of the pristine polyamide membrane. Furthermore, there is a significantly lower water flux decline of the zwitterionic polyamide membrane compared to the pristine polyamide membrane in filtrating a mixed feed solution containing organic foulants, such as bovine-serum albumin, alginate, and natural organic matter. Kaner et al. [60] reported a novel self-cleaning, photo-responsive membrane, and found that the pre-deposited foulant could be removed from the membrane surface in response to UV or visible light irradiation while maintaining stable pore size and water permeance. This is because the hydrophobic spiropyran groups within the membrane are converted to hydrophilic zwitterionic merocyanine groups by irradiation with UV light, resulting in the release of adsorbed molecules and the full recovery of the initial water flux. From a thermodynamics viewpoint, the Gibbs free energy of the system first increases and then decreases in the process of adsorption, and the deterioration of foulants follows to a certain degree [61]. In other words, much more Gibbs free energy is needed for hydrophilic nanofiltration membrane surfaces adsorption of foulants than that of hydrophobic membranes [62]. Chang et al. [63] synthesized zwitterionic diblock copolymers, poly(11-mercaptoundecyl sulfonic acid)-block-poly(sulfobetaine methacrylate) (PSA-*b*-PSBMA) with different PSBMA and PSA lengths, via atom-transfer radical polymerization (ATRP). They grafted PSA-*b*-PSBMA onto a polycation brush modified membrane surface via an electrostatic interaction between oppositely charged groups, resulting in the formation of ion pairs in the membrane (Figure 7). Their antifouling performance was improved with increasing the content of ionic SA and zwitterionic SBMA units in the copolymers, wherein the membrane with the optimum composition of PSA block and PSBMA block exhibited strong resistance to non-specific protein adsorption and excellent blood-inert properties. All of these results demonstrate there is high fouling resistance and good biocompatibility for membranes containing zwitterionic ion pairs.

**Figure 8:**
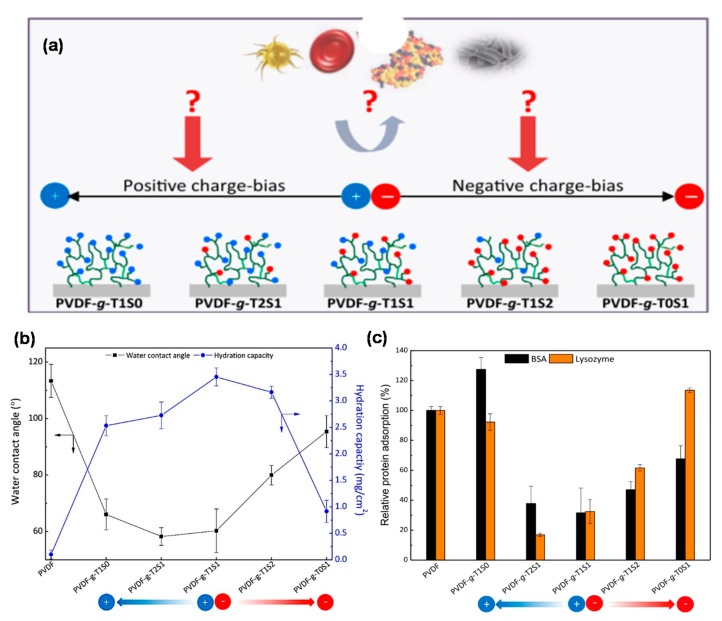
(**a**) Scheme of polyvinylidene Fluoride (PVDF) membranes coated with [2-(methacryloyloxy) ethyl] trimethylammonium (TMA) and sulfopropyl methacrylate (SA) monomers, before undergoing plasma polymerization. By carefully choosing the initial monomer ratio, the final surface charge can be controlled. Therefore, it should be possible to regulate the interactions with biofoulants from water, blood–proteins, bacteria, or blood cells, and lead to a low biofouling surface; (**b**) effect of plasma surface modification and mixed-charge molar ratio on the hydration properties of grafted layers; (**c**) effect of surface modification of PVDF membranes and surface charge-bias on resistance to nanofouling by bovine serum albumin (BSA) and lysozyme (LY) proteins (1 mL of either BSA or LY protein was added to the membrane sample with a diameter of 1.3 cm). Reproduced with permission from Reference [68].

As far as we know, the typical structure of zwitterionic materials includes both the positively and negatively charged moieties existing within the same polymer chain segment, maintaining overall charge neutrality. The balanced charge and minimized dipole of ion pairs were the key factors for the antifouling properties of zwitterionic materials, which can bind water molecules via electrostatic interactions and repulse charged foulants via electrostatic repulsion [64]. Bernstein et al. [65] modified polyamide membranes by grafting a zwitterionic and negatively and positively charged monomers on the membrane surface, respectively. It is found that the bacterial attachment on the membrane surface is dominated with the surface charge character and hydrophilicity. There is an enhanced deposition of negatively charged bacteria on the positively charged membrane surface, which is high for the pristine membrane, and much lower for the zwitterionic surface, while the negatively charged surface showed a long-range repulsion and negligible hysteresis. Hadidi et al. [66] systematically studied the antifouling behavior of zwitterionic membranes compared to charged and neutral membranes with similar pore sizes. They found that the extent of fouling was strongly affected by the hydrophobic and electrostatic interactions between proteins and membranes. The zwitterionic membranes showed excellent antifouling performance over a wide range of conditions with all proteins. Jiang’s group proposed that the antiparallel orientation of zwitterionic head, which is similar as the membrane lipids can adsorb high amounts of water molecules and render the materials resistant to fouling [67]. This means that when we use pseudo-zwitterionic materials (prepared from positively charged and negatively charged monomers), their charge distribution should be well controlled, otherwise the worse surface packing would decrease the amount of water molecules trapped and lead to a weak resistance to foulants. Venault et al. [68] modified poly(vinylidene fluoride) (PVDF) membranes with the copolymers of [2-(methacryloyloxy) ethyl] trimethylammonium (TMA) and sulfopropyl methacrylate (SA) to investigate the relationship between the membrane charge character and the membrane antifouling properties. It is convenient to control the charge of the membrane surface with the initial molar content for preparing either pseudo-zwitterionic membranes or positively/negatively charged membranes. As shown in Figure 8, there is a strongest electrostatic attraction between positively-charged membranes and negatively charged bovine serum albumin (BSA) on one hand, and negatively-charged membranes and positively charged lysozyme (LY) on the other hand, leading to serious protein adsorption. However, pseudo-zwitterionic membranes exhibit excellent resistance to different proteins, which is ascribed to the non-fouling power of ion pairs or to electrostatic repulsions when a charge bias is involved. At the same time, the pseudo-zwitterionic membranes have low-biofouling properties to *Escherichia coli*, as did the blood cells (platelets and erythrocytes). All of above results demonstrate that zwitterionic membranes with strong antifouling property and good biocompatibility have potential to be applied in either water treatment or blood filtration. 

The steric hindrance effect of zwitterionic polymer chains is another important factor for monitoring their membranes’ antifouling behavior. The molecular design of polymer brushes on the surface, e.g., the polymer chain length, polymer packing density, and polymer conformation, plays important roles in achieving excellent antifouling performances. Chang et al. [69] reported that the hydration of the membrane surface increased with increasing the zwitterionic polySBMA chain length. It is found that the existence of a thick zwitterionic polymer brush with sufficient hydration layers would provide strong steric hindrance, protecting the membrane from foulants. When the foulants come into contact with the membrane surface, first they would compress the zwitterionic brush and reduce the movability of the polymer chains, and the system Gibbs free energy would increase [70]. Thus, the polymer chains would likely recover from their swelling extension state, and stop the foulants from coming into contact with the membrane surface. Li et al. [71] grafted poly(zwitterionic 3-(methacryloylamino) propyl-dimethyl-(3-sulfopropyl) ammonium hydroxide) (PolyMPDSAH) on the surface of polyamide membranes to improve their antifouling property. The polyamide membrane was first activated with formaldehyde and phosphoric acid, and then grafted to PolyMPDSAH via free radical polymerization initiated by ceric ammonium nitrate. The grafting degree was increased from 0 to ~170.5 (μg/cm^2^) with increasing the MPDSAH monomer concentration (0, 3, 10, and 20 g/L), and as a result the hydrophilicity was enhanced and the antifouling performance was improved significantly. However, the hydraulic resistance of membranes was increased due to the larger amount of polyMPDSAH chains grafted on membrane surface, leading to a decrease in the water flux of grafted membranes and a corresponding salt rejection increase from ~92% to ~95%. This result indicates that there is a trade-off between water permeability and antifouling property in the surface grafting density. Thus, the zwitterionic polymer grafting density should be controlled to an optimum level to improve the membrane antifouling performance and have minimal impact on membrane separation performance. Sin et al. [72] proposed that the flexible zwitterionic polymer chains on that membrane surface have greater hydration capacity. They grafted polySBMA brushes from a dopamine-modified surface, with the zwitterionic chains packed in a loose state, resulting in a thick hydration layer due to the higher amount of free water molecules trapped within the space of the flexible polymer chains. This augmented the interfacial hydration capacity and induced a stronger steric hindrance effect for resisting foulant contact and adsorption on the membrane surface.

## 3. Fabrication of Nanofiltration Membranes Containing Ion Pairs

### 3.1. Nanofiltration Membranes Prepared with Oppositely Charged Polyelectrolytes

#### 3.1.1. Multilayer Polyelectrolyte Membranes 

Nanofiltration membranes can be prepared by the alternate LBL deposition of oppositely charged polyelectrolytes on top of a substrate layer, for example, exposing the positively charged substrate layer to a negatively charged polyelectrolytes solution, followed by rinsing to remove excess and weakly adsorbed polyelectrolytes. The surface charge character is reversed to a negative charge after the first adsorption step, and then the substrate layer is exposed to a positively charged polyelectrolytes solution, again followed by rinsing. This process is repeated several times to obtain a polyelectrolyte nanofiltration membrane (PENM) with the desired structure and thickness. The electrostatic interactions, hydrophobic interactions, and entropy gain play important roles in the LBL process for PENM preparation [73,74]. Schlenoff et al. proposed the charge overcompensation mechanisms, as there are the intrinsic and extrinsic charge overcompensations and competitive ion pairs in PENMs [75,76]. Ion pairs within the oppositely charged polyelectrolyte chains or some of polyelectrolyte segments is termed ‘intrinsic’ charge compensation, whereas ion pairs with charged counter-ions is termed ‘extrinsic’ compensation. Those ion pairs produced in PENMs are influenced by various parameters, such as the polyelectrolyte type, solution ionic strength, pH, solvent, and temperature. Thus, these would affect the formation, growth, structure, stability, and properties of PENMs, leading to a change in the nanofiltration performance of PENMs. 

**Figure 9:**
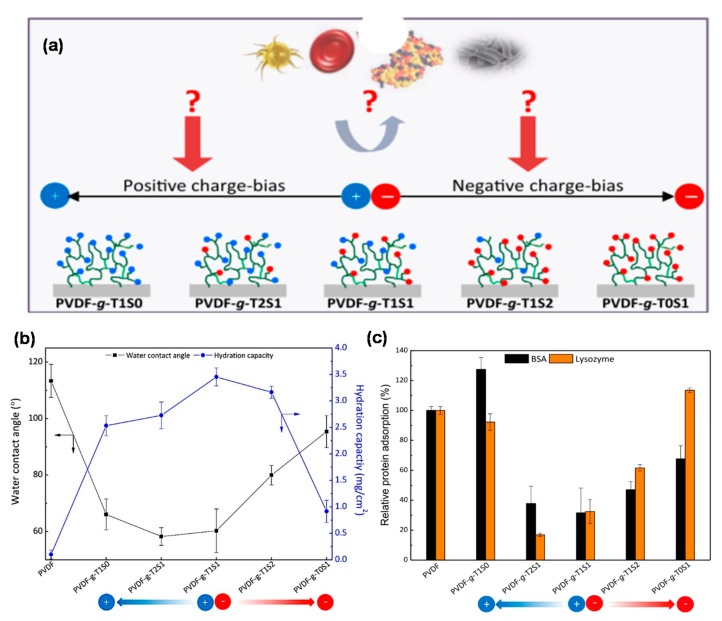
Chemical structures and abbreviations of common polyelectrolytes.

The choice of suitable polyelectrolytes is most important for PENM formation (chemical structures and abbreviations of some common polyelectrolytes are shown in Figure 9), which will influence the PENM growth and properties, such as the thickness, hydrophilicity, porosity, stability, and mechanical properties. The LBL PENMs formation depends on the strength of the ion pairs between oppositely charged polyelectrolytes. When the electrostatic interaction of ion pairs within PENMs is strong, there is mainly intrinsic charge compensation, and the LBL PENMs are built up following a linear growth mode. In contrast, the weak interaction of ion pairs within oppositely charged polyelectrolytes will lead to an exponential growth of LBL PENMs with both intrinsic and extrinsic charge compensations. In a previous study, Jin et al. [40] prepared PENMs by the LBL method with PVA and PVS on porous supports. A relatively high deposition number (60 bilayers) was chosen to make the polyelectrolyte multilayer dense and free of defects for separating. The salt rejection of PVA/PVS PENM increases in the series 1,1- < 1,2- < 2,1- ≤ 2,2-electrolyte, while the water permeability of the PENMs is relatively lower, at 0.1 L m^−2^ h^−1^ bar^−1^. Miller et al. [77] used a variety of oppositely charged polyelectrolytes to prepare LBL PENMs, and their nanofiltration performance could be optimized by varying the constituent polyelectrolytes. The rejections of PENMs to glycerol, glucose, and sucrose decreased in the order of PSS/PAH > PSS/PDDA > PSS/chitosan (CS) > HA/CS, which is also the decreasing order of polyelectrolyte charge densities (Table 1). This result suggests that the choice of polyelectrolytes with high charge densities will produce a heavily ionically cross-linked PENMs with high rejection of neutral molecules. In addition, they found that salt rejections of PENMs are mainly dominated by the composition of the membrane outer layer [78]. For example, CaCl_2_ rejection increases from approximately 86% to 96% after the deposition of a terminating PAH positively charged layer, while the opposite trend occurs for Na_2_SO_4_. In general, electrostatic (Donnan) exclusion plays a major role in salt rejection of the nanofiltration process, thus divalent-ion rejection increases when the charge of the membrane outer layer has the same sign as the divalent ion being rejected. Hong et al. [79] also showed that the nanofiltration performance of PENMs can be tuned by varying the constituent polyelectrolytes and LBL numbers. The solution flux of PENMs decreased in the order of PSS/PDDA, PSS/PAH > PAA/PDDA >> PAA/PAH, while the SO_4_^2−^ rejection was also highest for the PSS/PDDA system, making this PENM suitable for Cl^−^/SO_4_^2−^ separations. For example, (PSS/PDDA)_3_PSS PENM with the appropriate preparation conditions show a 96% rejection of SO_4_^2−^ and a Cl^−^/SO_4_^2−^ selectivity of 26. The solution flux 23.4 L m^−2^ h^−1^ bar^−1^ which is about three-fold higher than that of commercial nanofiltration membranes. Furthermore, they investigated the influence of deposition layer numbers on the membrane charge character and salt selectivity of PSS/PDDA PENM [50]. It is found that the thickness of PENMs increased and the magnitude of ζ potential decreased with the number of bilayers deposited, and both the Cl^−^/SO_4_^2−^ selectivity and water flux decreased when the bilayer number is higher than 4.5. The authors pointed that the variation trends in ζ potentials and ion selectivity with increasing number of bilayers are consistent with the exponential growth mode, where parts of polycations diffused to the surface of films to complex with polyanions from the solution.

**Figure 10:**
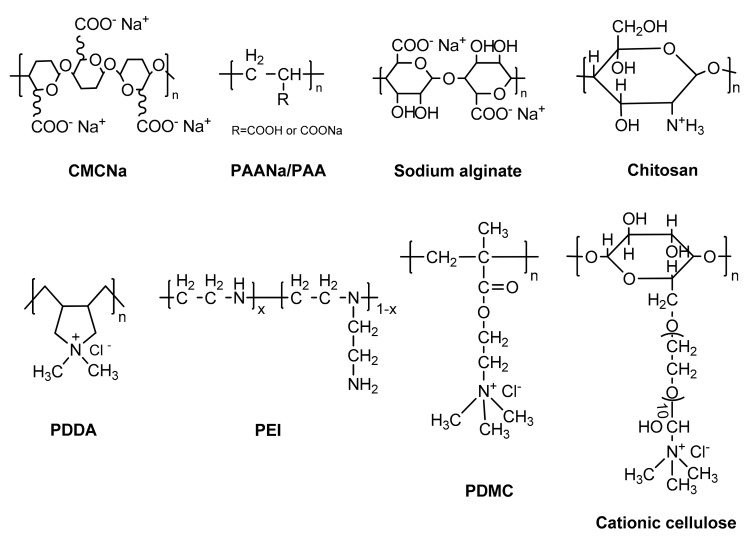
Effect of salt addition towards polyelectrolyte chains and thin film thickness (the NaCl concentration increases from 0 to 1.0 M). Reproduced with permission from Reference [87].

Nanofiltration membranes are usually utilized for desalination, drinking water purification, and waste water treatment, thus a good separation performance of membranes is required in feed solutions containing salts [80,81,82]. The effect of salt concentrations on LBL PENM preparation and separation performance has been studied in many works. Lu et al. [83] prepared LBL PENM with five bilayers of PSS/PAH on porous alumina supports. The membrane had a solution flux of 7.4 L m^−2^ h^−1^ bar^−1^, and exhibited 95% rejection of MgCl_2_ along with a Na^+^/Mg^2+^ selectivity of 22. The magnitude of the positive surface charge could be increased with increasing the Mg^2+^ concentration in the feed solution or when the outer polycation layer was formed in a high ionic strength solution, leading to a high separation performance for mono-/divalent salts. Ahmadiannamini et al. [84] fabricated LBL PENM with PDDA and SPEEK on hydrolyzed polyacrylonitrile (PAN-H) supports. It is found that both the thickness and water flux of PDDA/SPEEK PENMs increases with increasing the NaCl concentration from 0 to 0.5 M NaCl in the depositing solutions. This is because the polyelectrolyte chains stretch out to form a more linear structure in a weak ionic strength solution, resulting in thinner and denser films. On the other hand, with increasing the ionic strength of the deposition solutions, the electrostatic screening of polyelectrolyte chains increases, resulting in the formation of more coiled and loopy structures. Moreover, the decreased electrostatic interaction between polyelectrolyte chains will make the film growth change from a linear mode to a more dynamic exponential growth mode, since the extrinsic charge compensation becomes more important [85,86,87]. Thus, the polyelectrolytes deposited in a higher ionic strength solution will lead to weak attraction ion pairs, hence resulting in a thicker and looser membrane structure (Figure 10). Hong et al. [88] used the PSS/PDDA PENM to separate monovalent ions Cl^−^/F^−^, because fluoride is a harmful ion for health and its content in drinking water should be strictly controlled. (PSS/PDDA)_4.5_ LBL PENM exhibits a higher than 3 selectivity for Cl^−^/F^−^ and Br^−^/F^−^, and has a more than three-fold higher solution flux than those of NF 270 and NF 90 commercial membranes. The solution flux and ion selectivity were essentially constant with different feed concentrations, indicating that the separation performance of PENMs is stable in a certain range of feed salt concentrations. 

**Figure 11:**
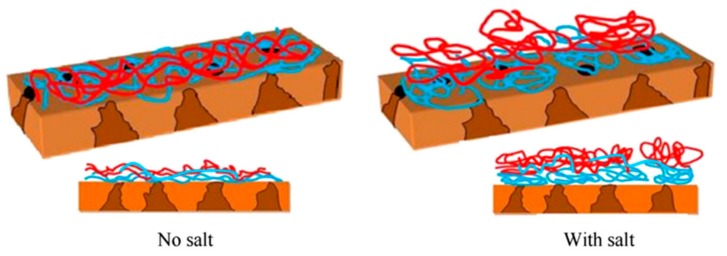
Graphical representation of layer by layer (LBL) deposition of polyelectrolyte nanofiltration membranes (PENMs) on hollow fiber ultrafiltration (UF) membrane support (top layer), retention of different micro-pollutants at pH 5.8 by PENM membranes with 6 (-) bilayers of poly(allylamine)/ poly(acrylic acid) (PAH/PAA) (prepared with different combinations of pH (6.0/6.0), (6.0/3.5), and (3.5/3.5) and ionic strength of 50 mM with 0.1 g L^−1^ polymer). Conditions for filtration experiments were: pH 5.8, Re ≈ 3100, and the applied trans-membrane pressure (TMP) of 1.8 bar (bottom layer). Reproduced with permission from Reference [48].

The charge density and structure of weak polyelectrolytes can be tuned by varying the pH of solutions [89,90], thus the nanofiltration performance of the obtained LBL PENMs can be adjusted by varying the pH of the polyelectrolyte solution or the filtration solution. Park et al. [91] chose weak polyelectrolytes, e.g., cationic PAH and anionic PAA, to prepare the PENMs by electrostatic the LBL and thermal cross-linking methods. It was observed that the cross-linked (PAH pH 7.5/PAA pH 3.5) n = 10,20 multilayers had a higher ion rejection compared to other multilayers. They claimed that there are amounts of unbound and uncharged groups for (PAH pH 7.5/PAA pH 3.5) PENMs, which would be converted to freely charged groups in the filtration of salt solutions. Shi et al. [92] prepared composite polyelectrolyte multilayer (C-PEM) membranes with (CS/PSS)_3_ multilayers (the middle layer, ML) and (PAH/PSS)_2_ multilayers (the top layer, TL) on hydrolyzed polyacrylonitrile (PAN) ultrafiltration membranes. Because the ionization degree of weak polyelectrolytes like PAH and CS could be tuned by pH, the nanofiltration performance could be adjusted by the feed pH value. It is found that the water flux underwent a drastic increase when the feed pH value was switched from neutral (3.7 L m^−2^ h^−1^) to basic (7.6 L m^−2^ h^−1^). In basic aqueous solution, the –NH^3+^ groups within CS and PAH molecules could be partially converted into –NH_2_ groups, and the interaction between polycations (CS and PAH) and PSS was reduced. Moreover, the left negatively charged groups increased the repulsive force of polyelectrolytes, resulting in a looser ionic cross-linked structure and higher flux. PENMs have been fabricated with the dynamic self-assembly of poly(4-styrenesulfonic acid-co-maleic acid) sodium salt (PSSMA) containing both strongly and weakly ionized groups, with PAH and PSS on modified PAN ultrafiltration membranes [93]. Because the charge density and conformation of PSSMA changed at different feed pH levels, the [PAH/PSS]_1_ PAH/PSSMA membrane prepared at pH 2.5 showed a higher rejection and larger flux (91.4% Na_2_SO_4_ rejection 14.3 L m^−2^ h^−1^ bar^−1^ solution flux) than those of the membrane prepared at pH 5.7. PENMs comprised of weak polyelectrolytes PAH and PAA were prepared on top of a hollow fiber ultrafiltration support (Figure 11). The charged character and ionic cross-linking density of the membrane was controlled by the pH of the coating solution. The separation performance of PENMs was dominated by both the size exclusion effect and Donnan exclusion effect. The PENM prepared at pH 6 showed a high micro-pollutant retention (60–80%) with a low ion retention [48]. In contrast, the PENMs containing strong ion pairs had good separation performance for amino acids without adjusting the feed pH value [94]. Seven bilayers of PSS/PAH were deposited on porous alumina, allowing selective transport of glycine from a mixture solution of glycine, l-alanine, l-serine, l-glutamine, and l-lysine. The selectivity of glycine over l-glutamine was about 50 with a solution flux of 12.2 L m^−2^ h^−1^ bar^−1^ at a pH of about 6.1 without adjustment. 

**Figure 12:**
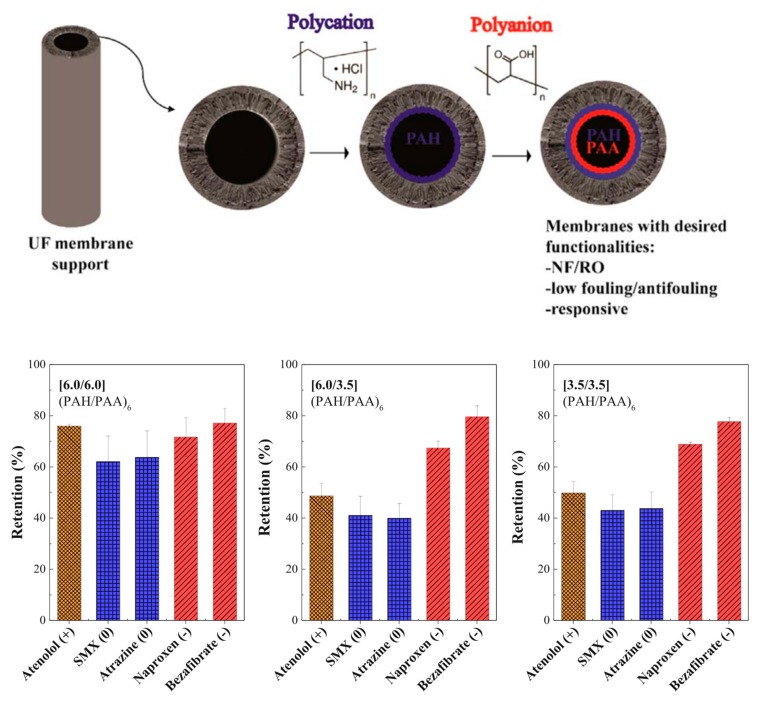
Schematic representation of the LBL preparation of a PEMM based on a linear cationic polymer (PDDA) and an anionic hyperbranched polyelectrolyte (HPE) sulfonated poly(aryleneoxindole) on a negatively charged support (hydrolyzed-polyacrylonitrile). Reproduced with permission from Reference [101].

With the development of nanofiltration technology, it has been applied in many fields such as substances separation and purification in petrochemistry, food chemistry, catalysis, and pharmaceuticals manufacturing, etc. [95,96,97]. Thus, nanofiltration membranes are required to be reliable and have good separation performances in organic solvent, referred to as solvent resistant nanofiltration (SRNF) or organic solvent nanofiltration (OSN). LBL PEMMs are considered to be very suitable for SRNF applications since their chemical structure and cross-linking density could be easily tuned, and polyelectrolyte films have a high permeability and good selectivity for charged molecules. Additionally, the insolubility of ionic cross-linked polyelectrolytes in most organic solvents renders them stable for SRNF. The separation performance of SRNF membranes is usually governed by the sieving effect, Donnan effect, and affinity effect. Li et al. [37] first prepared PENMs with PDDA and SPEEK for the SRNF applications. These PENMs showed a high rejection of charged dye molecules (>90% rejection of Rose Bengal) due to Donnan exclusion and different flux for different solvents such as isopropanol (permeance of 0.50 L m^−2^ h^−1^ bar^−1^), THF (permeance of 8.82 L m^−2^ h^−1^ bar^−1^), and DMF (permeance of 0.05 L m^−2^ h^−1^ bar^−1^). Later, Ahmadiannamini et al. [98] prepared PENMs with PDDA and PAA, of which the thickness and separation performance could be tuned with different NaCl concentrations and pH values in the deposition solutions. The obtained membranes showed good chemical stability and more than 90% retention for Rose Bengal in the filtration processes of aprotic solvent THF. In another work, they also investigated the SRNF performance of PVS/PSS PENMs with different polyanion types and cationic counter ions [99]. The membranes prepared with the H^+^-form of the polyanions showed higher solvent permeabilities and higher retentions for negatively charged Rose Bengal than those prepared with the Na^+^-form of polyanions because of their loopier structures and higher surface charges. These PEMMs have potential applications in SRNF; however, the time-consuming membrane preparation process and the relatively low permeability for organic solvent remain serious challenges [100]. Recently, Joseph et al. [101] fabricated an ultrathin single bilayer PENM based on the alternate deposition of a linear polyelectrolyte PDDA and sulfonated poly(aryleneoxindole) hyperbranched polyelectrolyte (HPE) on a hydrolyzed polyacrylonitrile (H-PAN) support for SRNF (Figure 12). The obtained (PDDA/HPE)_1_ PENM exhibited an ultrahigh solvent permeability (acetonitrile: 25 L m^−2^ h^−1^ bar^−1^) with a very high retention (94.5% rejection for acid fuchsin). This is because the HPE macromolecules with the 3D network structure can efficiently cover the H-PAN supporting membrane without filling the underlying pores, and the abundant terminal functional groups of HPE can cross-link with PDDA, resulting in a dense and thin selective layer with high separation performance. 

#### 3.1.2. Homogeneous Polyelectrolyte Complex Membranes 

**Figure 13:**
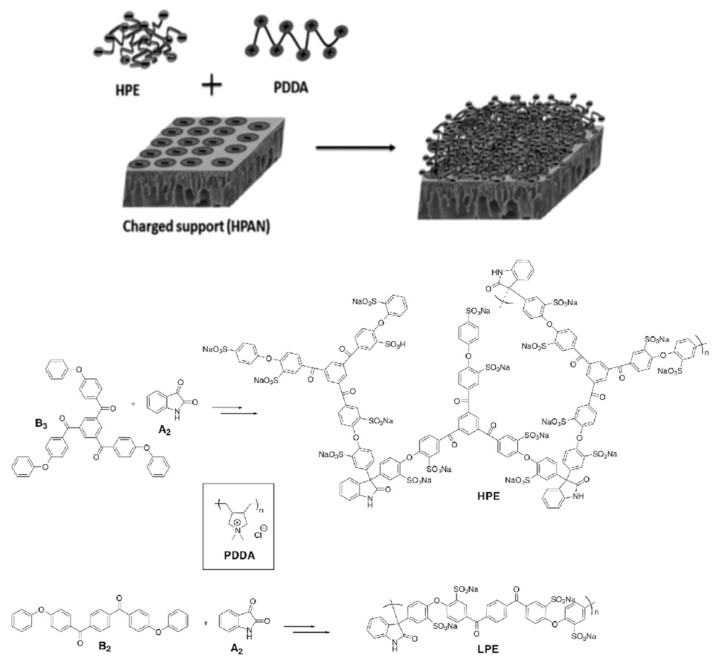
Schematic diagram for preparing polyelectrolyte complexes (PECs) and their composite nanofiltration (NF) membranes. Reproduced with permission from Reference [108].

Polyelectrolyte–polyelectrolyte complexes (PECs), formed by combining oppositely charged polyelectrolytes together via ionic interaction, are ideal polymeric materials for the fabrication of nanofiltration membranes. PECs, as a new kind of amorphous polyelectrolyte complex material, are insoluble in common solvents, which have some distinctive characteristics such as stable structures, high surface hydrophilicity, and tunable surface charge [17,102]. However, PECs are usually insoluble and infusible, and the difficulties to process the materials become the bottleneck of PEC membrane development. The acid blending method has been used to prepare PEC aqueous solution [103,104,105]; however, only limited polyelectrolyte pairs CS–PAA can be mixed in acid without precipitates or phase separation [106,107]. In addition, the excessive acid in the acid blending method may influence the PEC membrane structure and it does not absolutely resolve the processability problems of PECs. An and Ji et al. [108,109] proposed a surface coating method to prepare nanofiltration membranes with water-dispersible homogeneous polyelectrolyte complex (HPEC) materials. The HPEC bulk materials were prepared by the “protection-deprotection” method [110,111]. As shown in Figure 13, HPECs were synthesized with quaternary ammonium cellulose (QCMC) and sodium carboxymethyl cellulose (CMCNa). First, a weak polyacid CMCNa was dissolved in a certain concentration of acid solution, so that the ionization degree of CMCNa was suitable. Then, cationic polyelectrolyte QCMC solution was added to the anionic polyelectrolyte CMCNa solution to obtain bulk HPEC solids. It is worth noting that the HPEC solids with protected COOH groups can be re-dispersed in NaOH aqueous solution because the protonated COOH groups could be transferred into COONa groups again. Finally, HPEC nanofiltration membranes (HPECNFMs) were fabricated by the surface coating and chemical cross-linking of HPECs on polysulfone (PSF) ultrafiltration membranes. A weak anionic polyelectrolyte, e.g., CMC, PAA, hyaluronic acid (HA), sodium alginate (SA), etc., could be ionic cross-linked with various cationic polyelectrolytes, e.g., PDDA, poly(ethyleneimine) PEI, poly(2-methacryloyloxy ethyl trimethylammonium chloride) PDMC, CS, to make a membrane casting solution [17]. Compared with the LBL PENMs, it is more convenient to prepare HPECs and their nanofiltration membranes in a larger scale. In addition, the ionic cross-linking density and hydrophilicity of HPECs could be tuned by changing their constituent polyelectrolyte species or their mixing ratios. Moreover, the PECNFMs showed a good stability and antifouling capability for long-term operation. Thus, water-dispersible HPECs constitute a new family of materials that have great application potential for preparing nanofiltration membranes with good separation performance.

**Figure 14:**
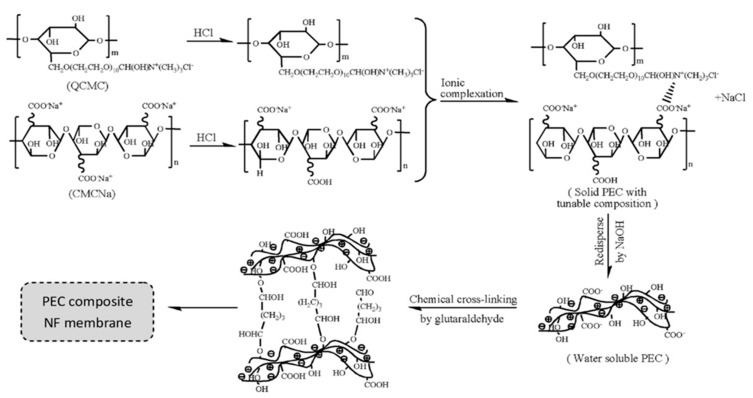
(**a**) SEM morphology for HPEC particles, (**b**) schematic diagram for water channel in HPECM, and (**c**) o-Ps lifetime distributions data for CMCNa and HPECM membranes. (PDF: Probability Density Function). Reproduced with permission from Reference [113].

There is an obvious improvement of water flux for HPECNFMs in comparison to the pristine CMCNa membrane [108,109]. Zhao et al. [112] found that the HPEC membranes are constructed of charged and needle-shaped HPEC nanoparticles. There are special water channels both between and inside HPEC particles. Positron annihilation lifetime spectroscopy (PALs) analysis revealed that both the orthopositronium (o-Ps) lifetime and free volume sizes of HPEC membranes are larger than those of CMCNa membranes [113]. The free volumes come from the spaces between HPEC nanoparticles and the spaces between polyelectrolyte chains inside HPEC nanoparticles. Additionally, it is found that there is long lifetime component (τ_4_) in HPEC membranes, which is attributed to the special aggregation structure of PECMs (Figure 14). It is speculated that the larger spaces between HPEC nanoparticles and their aggregations could provide additional channels for water molecules transporting through the membrane, while the “ion pairs” charged character would repel ions from the membrane. Thus, HPECs are fascinating materials for the construction of high performance nanofiltration membranes. HPEC nanoparticles associated with hydrophilic charged groups such as sulfate and sulfonate groups would further enhance the water permeability and antifouling properties of the obtained membranes [114]. An et al. [115] prepared a novel type of HPEC nanoparticle by using CS and dextran sulfate sodium as starting materials, and the sulfated HPEC membranes (SHPECMs) were prepared by solution-casting and the glutaraldehyde cross-linking method. It is revealed that the intrinsic aggregation structure combined with numerous sulfate groups increases both the packing density of polymeric chains and the membrane surface hydrophilicity, endowing SHPECMs with high water flux and solute selectivity. The selectivity of SHPECMs for NaCl/Na_2_SO_4_ and NaCl/methyl blue dye is 13.1 and 850.0, and the water permeability is 6.71 L m^−2^ h^−1^ bar^−1^, which is 2.3 times higher than that of the pristine sulfated CS membrane. Zhao and coworkers [116] reported a facile route to synthesize nanoporous PEC membranes (NPCM) with poly[1-cyanomethyl-3-vinylimidazolium bis(triuoromethanesulfonyl)-imide] (PCMVImTf2N) and PAA. The NPCM was prepared from a physically blended film of those two oppositely charged polyelectrolytes. After immersing the film in aqueous NH_3_ solution, electrostatic complexation and nanopores were produced. Finally, the membrane possessing three-dimensionally interconnected nanopores was obtained. Recently, Yang et al. [117] used a similar method to fabricate free-standing PEC nanofiltration membranes. They synthesized negatively charged copolymers with acrylic acid and acrylonitrile. The acrylic acid segment in the copolymers was used to form an ionic complex with imidazolium-based polycations, PCMVImTf2N, and a negatively charged PEC film with nanopores was obtained. This film had excellent mechanical properties (with a tensile strength of 6.7–23.7 MPa and an elongation at break of 15–59%), and exhibited moderate rejection to salts in the order of Na_2_SO_4_ > NaCl > MgCl_2_, as well as high rejection to methyl orange (>99.9%). As HPECMs show good substance separation performance, various of polyelectrolytes and charged macromolecules can be incorporated into HPECs. However, minimizing the thickness of HPECMs in the sub-micron scale and precisely controlling the size of nanopores in HEPCMs is crucially important for improving their nanofiltration performance.

**Figure 15:**
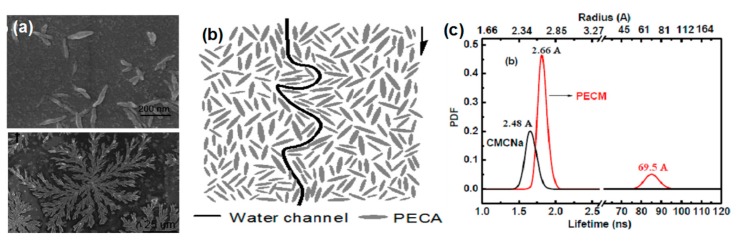
Schematic diagram for the mechanism of in-situ ionic cross-linking of PEC-NFMs and HPEC-NFMs. Reproduced with permission from Reference [122].

Mixed matrix membranes (MMMs) have become a hot research topic, as it is usually required for nanomaterials to be well-dispersed in membrane casting solution, have good compatibility, and strong interaction with the polymeric matrix. HPECs are the alternative candidates for the preparation of nanocomposite membranes, wherein nanomaterials could first be pre-dispersed in the component polyelectrolyte solution and incorporated into the HPEC matrix during the ionic complexation. Subsequently, the obtained HPEC nanohybrids are dispersed in a basic aqueous solution to fabricate their nanocomposite membranes by the surface coating method. Silica (SiO_2_) [118], multiwall carbon nanotubes (MWCNTs) [119,120], and graphene oxide (GO) [121] have been used as model globule, linear, and lamella nanofillers, thus improving the mechanical properties and separation performances of HPEC nanohybrid membranes. An et al. [122] prepared HPEC/MWCNT hybrid nanofiltration membranes via in situ ionic cross-linking between SA, PEI, and MWCNTs (Figure 15). It is found that the surface charge character of HPECNFMs can change from negative to positive with increasing the PEI content, comprising a simple and effective approach for fabricating positively charged nanofiltration membranes. Moreover, the water flux of HPECNFMs was enhanced from 13.4 to 27.0 L m^−2^ h^−1^ with increasing the MWCNTs content, and the MgCl_2_ rejection was maintained at around 93.5% (testing with 1 g L^−1^ aqueous MgCl_2_ solution at 25 °C and 0.6 MPa). Through the incorporation of MWCNTs ionic cross-linked with the –NH_3_^+^ groups of PEI, there is a strong interaction between MWCNTs and the HPEC matrix, thus improving both the mechanical strength and nanofiltration performance of HPEC hybrid membranes. Recently, Wang et al. [123] adopted the acid “protection-deprotection” method to fabricate HPECs by using CMCNa and PDDA. GO was added to the acidified CMCNa solution, the corresponding HPEC-GO was obtained, and then it was dispersed in a basic aqueous solution and casted onto a PVDF microporous substrate. The authors claimed that GO in the HPEC matrix mainly prevented the excessive aggregation of polymeric chains and increased the membrane density due to the ionic cross-linking effect. Thus, a high GO content in HPEC membranes could increase the mass transfer resistance, which then increases the salt rejection and decreases the water flux. 

### 3.2. Nanofiltration Membranes Prepared with Zwitterionic Materials

#### 3.2.1. Surface Grafting Zwitterionic Polymer Membranes

**Figure 16:**
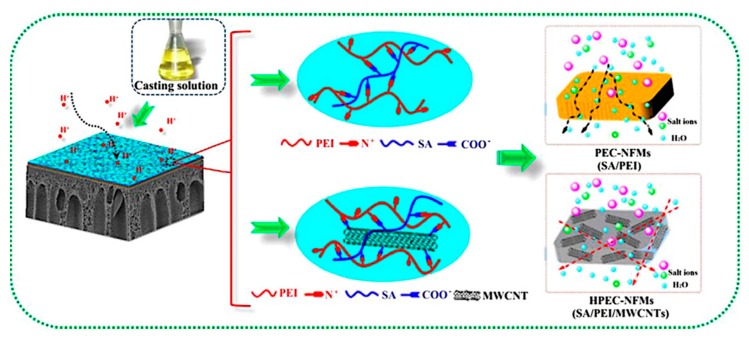
Representative, not comprehensive, summary of surface grafting zwitterionic polymers research: the reaction process for the activation of amide groups and graft polymerization of MPDSAH on the surface of polyamide membrane; schematic illustration of the improved UV-induced polymerization of poly(sulfobetaine methacrylate) onto MPPM; ATRP graft polymerization of MPC on a polyamide membrane. Reproduced with permission from References [71,131,138].

In order to enhance the hydrophilicity and antifouling properties of membranes, much research has been conducted to anchor zwitterionic polyelectrolytes onto membrane surfaces by the surface grafting method [124] (Table 2). Most of the reported works describe the modification of membrane surfaces via “grafting from” with zwitterionic monomers [125], while rare works adopted the “grafting to” with end-functionalized zwitterionic polymer chains. As shown in Figure 16, zwitterionic polymers can be grafted from the membrane surface by the conventional free radical graft technique, radiation graft approaches, “living”/controlled graft polymerization, and so on. Thus, the antifouling performance of the resultant membranes (to inorganic fouling, organic fouling, and biological fouling) was improved, and ultimately the separation performance was greatly enhanced. The conventional free radical graft technique is a facile and effective approach to prepare composite membrane or modify the membrane surface with improved permselectivity and antifouling properties. Liu et al. [126] prepared zwitterionic membranes by free radical polymerization between glycidylmethacrylate (GMA) and acrylic acid (AA) monomers, resulting in the ring-opening of epoxide and quaternary amination with trimethylamine hydrochloride. Because both anionic and cationic groups simultaneously exist in the membrane matrix, there is high net charge density, which facilitates the transport of anions and cations through the membrane. Bernstein et al. [65] modified polyamide membranes with sulfobetanine zwitterionic monomers by concentration polarization-enhanced radical graft polymerization. With a low grafting degree, the water flux of membrane decreased a little (by 20–40%) and the salt rejection did not change, yet the membrane surface become more hydrophilic and less negatively charged, thus the antimicrobial performance of the membrane was significantly improved. Recently, Li et al. [71] also modified the polyamide nanofiltration membranes with formaldehyde and then used the cerium (Ce (IV)) for inducing graft polymerization of zwitterionic 3-(methacryloylamino) propyl-dimethyl-(3-sulfopropyl) ammonium hydroxide) (MPDSAH) monomers, and the obtained membrane exhibited enhanced antifouling propert. The polyamide membrane could also be modified with zwitterionic polymer poly(4-(2-sulfoethyl)-1-(4-vinylbenzyl) pyridinium betaine) (PSVBP) via free radical polymerization initiated by a K_2_S_2_O_8_–NaHSO_3_ redox system [127]. It is found that there is an enhanced cleaning efficiency for the modified membrane because of the phase transition and conformation variation of the grafted PSVBP polymer chains, and as a result the deposited foulant layer loosens and can be removed from the membrane surface. Wang et al. [32] used K_2_S_2_O_8_/Na_2_S_2_O_5_ as redox initiator for the graft polymerization of *N*,*N*-dimethylaminoethylmethacrylater (DMAEMA) on commercial polyamide membranes (TE), followed by the quaternization reaction with 3-bromopropionic acid, resulting in a zwitterionic carboxybetaine methacrylate-modified membrane, TE–PCBMA. The grafting degree of zwitterionic polymers increases with increasing DMAEMA concentrations in the grafting solution. The water flux of the TE–PCBMA membrane increased 22.55% compared to that of the pristine TE membrane, while their salt rejections maintained the same. Moreover, there is a better resistance of TE–PCBMA membrane to nonspecific protein adsorption; after rinsing, the fluxes of BSA and lysozyme fouled TE–PCBMA membranes could be recovered to 92.11% and 95.19%, respectively.

Up to now, some radiation graft approaches, e.g., ultraviolet (UV) radiation graft, ozone-initiated graft, and plasma-induced graft, have been exploited for the fabrication of zwitterionic polyelectrolyte composite nanofiltration membranes [128,129]. Photosensitive membrane materials such as polysulfone, polyethersulfone, and polyamide can generate free radicals with UV radiation, and then zwitterionic polymers could be grafted onto the membrane surface. Tirado et al. [130] adopted UV-initiated graft polymerization to anchor zwitterionic polymers on commercial polyamide membranes. First the membrane sample was initiated with benzophenone (BPh), and then UV-grafted polymerized with 2-[(me-thacryloyloxy)ethyl] dimethyl-(3-sulfopropyl) ammonium hydroxide (SPE). The modified membrane had a much more hydrophilic and less negatively charged membrane surface, which resulted in a resistance to bacteria deposition. The hydrophilicity and antifouling properties of modified membranes is directly relevant to the grafting density of zwitterionic polymers, which is governed by photosensitizer concentration, zwitterionic monomer concentration, and UV irradiation intensity and time, respectively [131,132]. Ozone-initiated polymerization can also be used to graft zwitterionic polymers on membrane surfaces, wherein the peroxides are firstly introduced onto the membrane surface, then decomposed into free radicals which initiate the grafting of zwitterionic polymers from membrane surfaces [133,134]. Some hydrophobic membranes, e.g., polypropylene (PP) or polyvinylidene fluoride (PVDF) porous membranes, can be activated with ozone and grafted with hydrophilic polymers to modify or fabricate nanofiltration membranes. Additionally, with the plasma treatment, active atomic, ionic, and radical species can be implanted onto the membrane surface, and initiate grafting from the polymerization of zwitterions. The grafting density and the length of zwitterionic polymer chains can be tuned by different plasma parameters, such as the pressure, power, sample disposition, treatment time, monomer concentration, and so on [68,135]. Although these abovementioned graft methods have been successfully used to prepare zwitterionic membranes, there are still several disadvantages that should be overcome. For example, the efficiency of initiating free radicals is relatively low, and as a result there is a low grafting density of zwitterionic polymers. Simultaneously, the growth of polymer chains is not easy to control, which may lead to an uneven distribution of grafted zwitterionic polymers on the membrane surface. In addition, some radiation graft techniques may result in the degradation of the polymeric matrix membrane, and the special radiation equipment is usually high-cost not suitable for large-scale industrial production. 

“Living”/controlled graft polymerization has become an alternative method for grafting zwitterionic polymer on membrane surfaces. The ATRP process developed by Prof. K. Matyjaszewski et al. has been widely used to graft polymer chains or brushes onto membrane surfaces [136,137]. ATRP employs alkyl halide as an initiator with transition metal complex as a catalyst, and the number of initiators immobilized on the membrane surface usually determines the graft density of the zwitterionic polymers on the membrane surface, while the length of grafted polymer chains is dependent on the reaction time of ATRP. Saeki et al. [138] grafted zwitterionic phosphorylcholine polymer on a polyamide membrane using the ATRP method. The polyamide membrane was first prepared and modified with diethanolamine (DEA) via interfacial polymerization. The ATRP initiator, 2-bromoisobutyryl bromide (BIBB), was then immobilized on the membrane with the condensation reaction between DEA and BIBB. Finally, ATRP was carried out using 2-methacryloyloxyethyl phosphorylcholine (MPC) and immobilized BIBB. The MPC amount increased and the surface became smoother with increasing the grafting time. The MPC-grafted polyamide membrane showed a high anti-biofouling performance with maintaining its initial water flux. Recently, Zhang et al. [139] reported a salt-responsive polyamide membrane achieved by grafting poly(sulfobetaine methacrylate) (PSBMA) brushes on a membrane surface by ATRP. The 2-bromo-2-methylpropionyl bromide (BiBB) was first deposited on the membrane surface with dopamine, then the membrane was immersed in the SBMA isopropyl alcohol aqueous solution and initiated with a mixture of CuCl_2_ and tris(2-pyridyl methyl) amine (TPMA). With increasing the grafting time from 0 to 12 h, the grafting yield increased linearly from 0.23 to 0.83 mg/cm^2^, and the modified membrane exhibited superhydrophilicity and underwater superoleophobicity, as demonstrated by the water contact angle of 8° and underwater oil contact angle of 149°. The oil-adhesion force on the TFC-PSBMA membrane was significantly decreased to be 11.1 Μn, compared with the pristine TFC polyamide membrane (24.5 μN), while after cleaning with 0.5 M NaCl solution, the oil-adhesion force of the TFC-PSBMA membrane was shown to be ~0 μN. This is thought to be ascribed to the salt-responsive ability of the PSBMA brushes, which results in a lowest irreversible fouling. From previous studies, the ATRP method is found to conveniently tailor the graft density, chain length, and chemical composition of zwitterionic polymers on the membrane surface, and the membrane morphology and properties can be fine-tuned according to application requirements. However, the ATRP graft process is relatively complicated and not suitable for large-scale fabrication. Also, the transition metal used in the ATRP graft process is a concern for the human health and protection of the environment. 

#### 3.2.2. Interfacial Polymerization Zwitterionic Membranes

**Figure 17:**
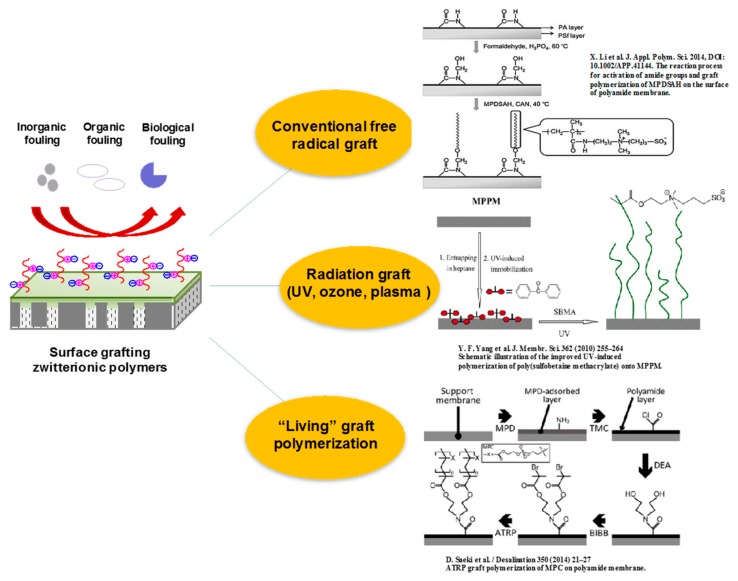
(**a**) Schematic diagram for preparing ZTFCMs. (**b**) Schematic model for water transport and antibacterial illustration of ZTFCMs. (**c**) Chemical structure of ZTFCMs. Reproduced with permission from Reference [144].

Since 1970, when the first polyamide membrane was prepared by interfacial polymerization, many types of polyamide thin film composite (TFC) nanofiltration membranes have been studied and employed in commercial desalination, water treatment, substances separation and purification, etc. [82,140]. It is required to design and synthesize various types of active monomers with unique functionalities and properties for optimizing membrane nanofiltration performances. Chiang et al. [141] proposed a new approach for preparing zwitterionic polyamide nanofiltration membranes containing carboxybetaine mimetic structures. The nanofiltration membranes were first prepared with diethylenetri-amine (DETA) and trimesoyl chloride (TMC) by the interfacial polymerization method, then they were further modified by iodopropionic acid and iodomethane through an *N*-alkylation reaction. The obtained membrane with a zwitterionic structure (simultaneously containing tertiary amine and propionic acid) exhibited excellent antifouling performance [138]. In order to simplify the preparation process and regulate the membrane performance, the synthesis of zwitterionic monomers for the fabrication of advanced polyamide membranes is desired. An et al. [33] firstly designed and synthesized a new type of zwitterionic amine monomer (AEPPS), which can take part in the interfacial polymerization for the preparation of zwitterionic TFC nanofiltration membranes. The zwitterionic monomer AEPPS was synthesized by the ring-opening reaction of *N*-aminoethyl piperazine with 1,3-propanesultone. Zwitterionic TFC membranes were prepared with piperazine (PIP), AEPPS, and trimesoyl chloride (TMC) by interfacial polymerization on a polysufone ultrafiltration membrane. With increasing the AEPPS content from 0 to 3.2 mol %, the water flux of the membrane increased from 23.4 to 43.1 L m^−2^ h^−1^ while K_2_SO_4_ rejection was maintained at around 97% (Table 2). Zwitterionic amine monomers (AEPPS) containing primary and secondary amine groups could also react with the residue acyl chloride groups of the nascent polyamide layer. As such, they constructed a zwitterionic modification layer on polyamide nanofiltration membranes, and both the water permeability and antifouling properties were further improved [142]. However, as the diffusivity and reactivity of AEPPS is relatively low, there is still a challenge to fabricate zwitterionic polyamide membranes with zwitterionic monomers directly [143]. With the adjustment of the preparation conditions and the addition of accelerants (±)-10-Camphorsulfonic Acid (CSA) and triethylamine (TEA) in the aqueous solution, the interfacial polymerization rate and cross-linking degree between AEPPS and TMC was increased, and zwitterionic polyamide membranes were successfully prepared [144]. There are many more zwitterionic groups on the membrane surface (the highest AEPPS content can reach 31.0 mol %), and the membrane becomes much more smooth and hydrophilic (Figure 17); as a result, it shows a stable and good separation performance for antibiotics and salts. Ma et al. [145] adopted a similar method to fabricate electro-neutral zwitterionic nanofiltration membranes at neutral pH. The zwitterionic amine monomer PEI-g-SBMA was synthesized by the Michael addition reaction with PEI and sulfobetaine methacrylate (SBMA). Then the PEI-g-SBMA/TMC membrane was prepared via interfacial polymerization on a polyethersulfone ultrafiltration (PES-UF) membrane. The membranes showed high dye/salts separation (solute rejection (90.6% for Orange GII, 50.4% for Na_2_SO_4_, 7.1% for NaCl) and good antifouling property against foulants. There is a good permselectivity and antifouling performance characterizing polyamide membranes prepared with zwitterions, which could be expanded to some other membrane processes such as reverse/forward osmosis and gas separation [34].

**Figure 18:**
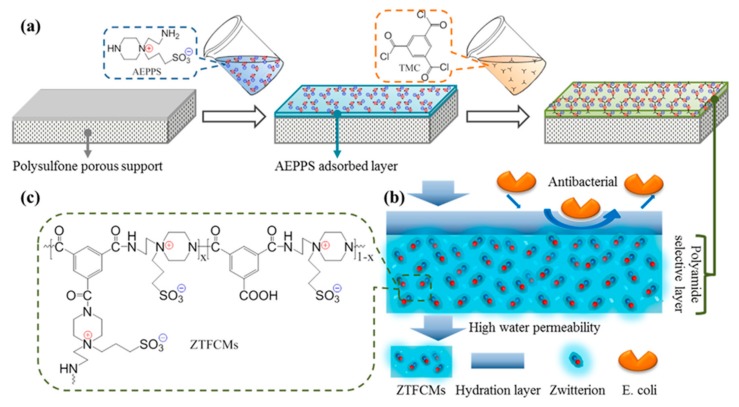
(**a**) Fabrication of ZPNPs and their thin-film nanocomposite (TFN) nanofiltration membranes; (**b**) Separation performances of TFC, TFC-CMCNa, TFN-PNP, and TFN-ZPNP3 membranes tested with pure water and 1 g L^−1^ aqueous Na_2_SO_4_ and NaCl solutions (pH = 7.0) at 25 °C and 0.6 MPa; comparison of the TFN-ZPNP3 membrane with various polyamide membranes reported in previous studies. Reproduced with permission from Reference [149].

In recent years, great progress has been made in the development of thin-film nanocomposite (TFN) nanofiltration membranes with various nanoparticles [146]. Polymeric micro/nanoparticles possessing greater flexibility for membrane formation, better compatibility with polymeric matrix, and easier tunability in particle structure have become a new class of nanomaterials for preparing TFN membranes [147,148]. Recently, An and Ji et al. [149] developed a new type of TFN nanofiltration membrane based on zwitterionic polyelectrolyte nanoparticles (ZPNPs) via interfacial polymerization. ZPNPs composed of zwitterionic copolymers (ZPE) and sodium carboxymethyl cellulose (CMCNa) were prepared through the ionic cross-linking method (Figure 18). The hydrophilicity and charged character of ZPNPs could be conveniently tuned by adjusting the zwitterionic group content and ionic cross-linking degree. The obtained zwitterionic TFN membrane exhibited an optimized nanofiltration performance, that is, both NaCl/Na_2_SO_4_ selectivity and water permeability increased from 22.7 to 28.4 and from 57.5 to 109.7 L m^−2^ h^−1^ MPa^−1^, respectively. In addition, zwitterionic nanogels (ZNGs) particles were synthesized with 3-dimethyl (methacryloyloxyethyl) ammonium propane sulfonate and 2-hydroxyethyl acrylate by surfactant-free emulsion polymerization [150,151]. With the incorporation of ZNGs into the polyamide membrane, the membrane surface became much more hydrophilic and negatively charged, resulting in high water permeability and good salt selectivity. Positron annihilation results demonstrate that the enhanced water permeability is mainly attributed to the increased hydrophilicity and the extra nano-cavities in zwitterionic TFN membranes, which provide preferential flow paths for water molecules. The high di-/monovalent salt selectivity was achieved by the strong electrostatic repulsion and the appropriate free volume within modified membranes. We thought that incorporating suitable organic particles into a compatible polymeric matrix represents an innovative approach to construct interfacial channel membranes for substance separation and water treatment applications.

Zwitterionic materials can also be used to modify inorganic nanomaterials to then fabricate hybrid TFN membranes. Chan et al. [152] prepared acylated CNTs which were then esterified using 3-dimethylamino-1-propanol, (CH_3_)_2_-N-C_3_H_6_-OH, followed by a ring-opening reaction of lactone, resulting in zwitterionic carboxylbetain group-modified CNTs. Zwitterion functionalized CNTs were deposited onto the PES support layer through vacuum filtration, then the semi-aligned CNTs layer was contacted with MPD and TMC solutions for interfacial polymerization, and TFN membranes containing zwitterionic CNTs were obtained. The water flux of the membrane was increased from 11.5 to 48.7 L m^−2^ h^−1^ with increasing the zwitterionic CNT content from 0 to 20 wt %. Recently, An et al. [153] fabricated a new kind of zwitterionic functionalized multi-walled carbon nanotubes (MWCNTs)/polyamide hybrid nanofiltration membrane; the modified MWCNTs were prepared by the co-deposition of dopamine and *N*-aminoethyl piperazine propane sulfonate (AEPPS) on MWCNT surfaces, after which AEPPS-PD@MWCNTs were mixed with piperazine (PIP) aqueous solution and interfacial polymerized with 1,3,5-benzenetricarboxylic chloride (TMC) to prepare hybrid a TFN nanofiltration membrane. The resultant membrane showed high water permeability, stability, and good separation capability for monovalent/divalent salts and monovalent salts/neutral organics. Zhu et al. [154] reported a surface zwitterionization of graphene oxide (GO) with grafting poly(sulfobetaine methacrylate) (PSBMA) via reverse atom transfer radical polymerization (RATRP), and a loose nanofiltration membrane GO-PSBMA/polyethersulfone (PES) was prepared via phase inversion. It was found that the water flux of the hybrid membrane was greatly enhanced with increasing the GO-PSBMA loading content, and exhibited good dye/salts separation performance. Moreover, the GO-PSBMA embedded membrane showed excellent antifouling performance (94.4% flux recovery ratio) and high mechanical strength (5.6 MPa tensile strength). 

#### 3.2.3. Surface Coating Zwitterionic Polymer Membranes

**Figure 19:**
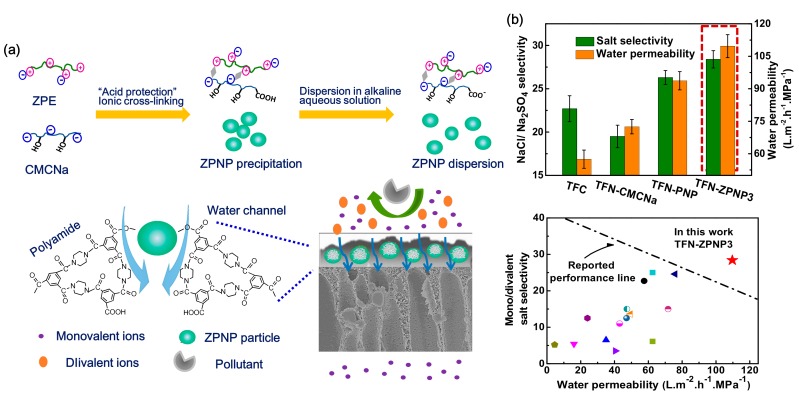
(**a**) Schematic diagram for preparing zwitterionic terpolymers PDHD and their composite nanofiltration membranes fabricated with surface coating and chemical cross-linking method; (**b**) Nanofiltration performance (MgCl_2_/NaCl selectivity/water flux) versus o-Ps lifetime and intensity of NFMs; (**c**) o-Ps lifetime distributions data for zwitterionic composite nanofiltration membranes (PDF = Probability Density Function). Reproduced with permission from References [155,156].

Surface coating is a convenient method for preparing composite membranes. The obtained membranes usually have a dense and smooth surface [97,124]. The most commonly used method is the traditional solution coating and cross-linking method. Zwitterionic polymers contain both anionic and cationic groups in polymer segments, thus there is strong intra-/interaction between the oppositely charged groups, resulting in a poor solubility in casting solution [18]. An and Ji et al. [155] designed and synthesized a novel type of water soluble zwitterionic terpolymer, which was composed of 2-methacryloyloxy ethyl trimethylammonium chloride (DMC), 2-hydroxyethyl acrylate (HEA) and 3-dimethyl(methacryloyloxyethyl) ammonium propane sulfonate (DMAPS). The hydrophilicity and charged density of PDHD terpolymers could be easily tuned by changing the monomer ratio in aqueous phase free-radical copolymerization. Zwitterionic composite nanofiltration membranes were prepared by surface coating and chemical cross-linking with PDHD and glutaraldehyde (GA) on polysufone ultrafiltration membrane. There is good salt resistance and strong antifouling properties for zwitterionic composite membranes because of the unique anti-polyelectrolyte behaviors of PDHD terpolymers and the formation of a “free water” hydration layer on the membrane surface. Furthermore, we used age–momentum correlation (AMOC) positron annihilation technology to investigate the microstructure of zwitterionic composite membranes [156]. The AMOC results showed that both the size and size distribution of nano-cavities in the selective layer increased with increasing the zwitterionic content, while the free volume varied inversely. The larger nano-cavities and smaller free volume is attributed to the higher water permeability and higher salt (divalent/monovalent) selectivity (Figure 19). In addition, zwitterionic copolymers made from 3-dimethyl(methacryloyloxyethyl) ammonium propane sulfonate (DMAPS) and 2-hydroxyethyl acrylate (HEA) could assemble into colloid particles in aqueous solution. They were coated and cross-linked with glutaraldehyde on a PSF-UF supporting layer to fabricate composite nanofiltration membranes [150]. The nanostructures of zwitterionic colloid particles and their membrane performances were adjusted by tuning the polymer composition, the acid concentration in the casting solution, and the pH value of the feed solution. Moreover, a kind of pH-responsive nanofiltration membrane was fabricated with carboxybetaine copolymers by a similar surface coating and chemical cross-linking method [47]. The zwitterionic copolymers were prepared with carboxybetaine methacrylamide and *N*-(Hydroxymethyl) acrylamide, and their membranes were shown to be tunable and reversible pH-responsive for organic molecules/salts separation. This responsive capability of zwitterionic nanofiltration membranes is highly desirable, as it would allow a single membrane to be used for a range of salts/molecules separation by fine-tuning the operation parameters such as temperature, ionic strength, and pH. 

**Figure 20:**
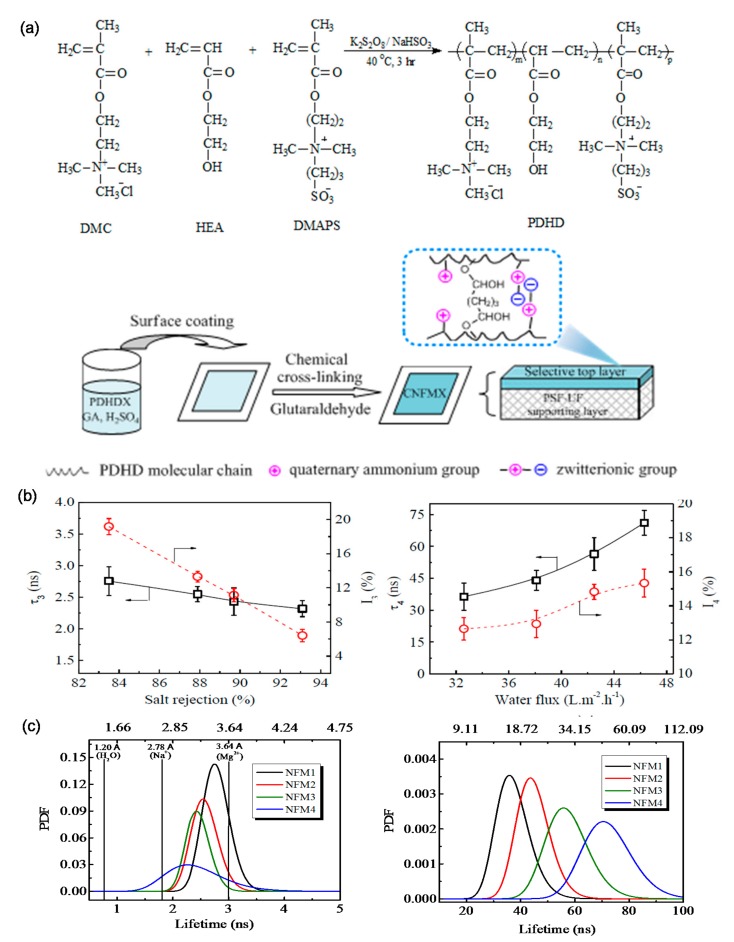
(**Left**) Schematic showing the self-assembly of zwitterionic amphiphilic random copolymers to form a network of water-permeable effective “nanochannels” and the interaction was formed between the selective layers of membranes with foulants (orange); (**Right**) TEM brightfield images of the self-assembled morphology of amphiphilic zwitterionic copolymers, exhibiting bicontinuous networks of zwitterionic (dark) and TFEMA (light) microphases. The insets show FFT of the images with the characteristic period shown on the arrow; (**a**) PT:SBMA, (**b**) PT:MPC, (**c**) PT:SB2VP, and (**d**) PT:SBB2VP exhibit similar characteristic periods of ~2.4 nm, yielding a zwitterionic channel size around 1.2 nm. Reproduced with permission from Reference [164].

The preparation of defect-free selective layers with sub ~100 nm thicknesses is difficult to achieve by conventional surface coating methods. The LBL assembly method could conveniently control the layer growth and chemical structures by the deposited time and condition. In Kharlampieva’s work [157], zwitterionic polymers were deposited on the substrate by the LBL assembly method, and under acidic conditions, weak zwitterionic polymers were incorporated into traditional multilayers. Grooth et al. [158,159] introduced poly *N*-(3-sulfopropyl)-*N*-(methacryloxyethyl)-*N*,*N*-dimethylammonium betaine (PSBMA) into the multilayers of PDDA/PSS on a sulfonated poly(ether sulfone) hollow fiber ultrafiltration membrane. As the zwitterionic polyelectrolyte multilayers are stimuli-responsive toward the ionic strength of the filtration solution, the water permeation increased up to 108% from 0 to 1.5 M NaCl. This is due to the “anti-polyelectrolyte effect” of zwitterionic polymers, thus the swelling degree of zwitterionic layers increased with increasing the salt concentration and the flux also was enhanced. Furthermore, the membranes terminated with PSBMA have a high retention for both positively and negatively charged micropollutants, which cannot be explained by Donnan exclusion, but instead dielectric exclusion is the dominating rejection mechanism. Initiated chemical vapor deposition (iCVD) is a polymer deposition technique [160], which is particularly suitable for membrane preparation. The film thickness can be controlled at the nanometer level and the membrane surface roughness is very low. Yang et al. [161] first reported antifouling, surface-attached zwitterionic ultrathin (30 to 100 nm) films synthesized by the iCVD method. Poly[2-(dimethylamino)ethyl methacrylate-co-ethylene glycol dimethacrylate] (PDE) thin films were constructed via iCVD and then reacted with 1,3-propane sultone to obtain the zwitterionic film. After that, they prepared another type of pyridine-based zwitterionic-modified polyamide membrane by the iCVD method, and these membranes showed improved resistance against a variety of molecular foulants and enhanced tolerance to chlorine [162]. Recently, Bengani et al. [163] prepared a new type of nanofiltration membrane via zwitterionic copolymer self-assembly on a porous supporting membrane. These zwitterionic copolymers are self-assembled into bicontinuous nanodomains, where the zwitterionic hydrated domains act as a bicontinuous network of “nanochannels” for solute and water permeation, and the hydrophobic segments construct the “channel walls” (Figure 20). The result showed that the self-assembled membrane with around a 1-nm pore size had a permeance as high as 8.4 L m^−2^ h^−1^ bar^−1^, over three times that of commercial membranes with a similar molecular weight cut-off (MWCO). Furthermore, they prepared a series of random copolymers with hydrophobic monomer 2,2,2-trifluoroethyl methacrylate (TFEMA) and four zwitterionic monomers (sulfobetaine methacrylate, sulfobetaine 2-vinylpyridine, sulfobutylbetaine 2-vinylpyridine, and 2-methacryloyloxyethyl phosphorylcholine) by free radical polymerization [164]. The self-assembled membrane prepared from the copolymer with 2-methacryloyloxyethyl phosphorylcholine was the most hydrophilic and showed the highest water permeance and strongest fouling resistance, showing no measurable flux decline throughout a 24-h protein fouling experiment.

**Figure 21:**
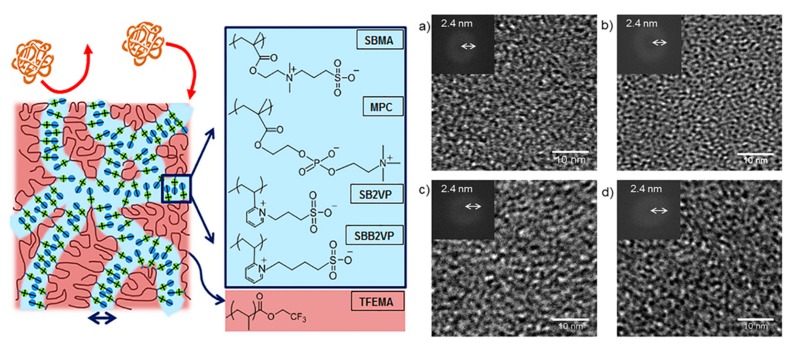
(**Top-line**) Co-deposition of PDA/PSBMA Triggered by CuSO_4_/H_2_O_2_; (**Bottom-line**) (**a**) Dynamic BSA filtration and (**b**) protein (BSA, Hgb, and Lys) adsorption quantity of the nascent and PDA/PSBMA-coated PPMMs. Reproduced with permission from Reference [169].

Biomimetic adhesion is inspired by the strong adhesion of mytilus edulis foot. Since 2007, several catechol derivatives have been developed for surface modification due to their strong and universal adhesion ability, through a simple and facile deposition process [165,166]. Generally, the interactions between coating layers and pristine membranes are nonspecific interactions, while with the biomimetic adhesion of zwitterionic polymers could be stably anchored on the membrane surface. The deposition process is more simple and controllable, and can be adjusted by varying the pH, concentration, deposition time, temperature, and atmosphere. Azari et al. [167] incorporated redox functional amino acid 3-(3,4-dihydroxyphenyl)-l-alanine (l-DOPA) onto commercial polyamide membranes to create a zwitterionic antifouling surface. After a 12-h l-DOPA modification, the membranes’ hydrophilicity was enhanced greatly because the poly l-DOPA, comprising “ion pairs”, could strongly interact with water via ionic–dipole interactions. Thus, the water flux of the modified membrane was increased, and an improved antifouling performance to BSA and alginic acid sodium foulants was achieved. The zwitterionic membrane showed less water flux decline than the pristine polyamide membrane, where the water flux recovery of 24-h-modified membrane reached up to 98% after cleaning with water. Zhou et al. [168] modified the membrane surface with zwitterionic polymer poly(sulfobetaine methacrylate) (PSBMA) via a simple one-step co-deposition process based on the self-polymerization and high adhesion properties of dopamine. Comparing with the UV-induced grafting method, the utilization efficiency of PSBMA increased more than 10 times up to be 9.13 wt %. The one-step co-deposition of PDA with zwitterionic polymers is becoming a convenient and controllable strategy for the fabrication of antifouling membrane surfaces. However, the co-deposition process is very slow, generally requiring 10 h or even several days, which is a big problem for membrane preparation. Recently, Xu et al. [169] utilized CuSO_4_/H_2_O_2_ as a trigger to accelerate the production of reactive oxygen free radicals, which can rapidly initiate the copolymerization of dopamine and zwitterionic poly(sulfobetaine methacrylate) (PSBMA) and greatly improve their co-deposition rate. The modified membrane showed an outstanding antifouling property (Figure 21), which could be maintained in acid and alkali solutions as well as in organic solvents. This work indicates that biomimetic adhesion zwitterionic materials provide a facile and efficient way to fabricate durable antifouling membranes. 

### 3.3. Charged Mosaic Membranes Containing “Ion Pairs”

**Figure 22:**
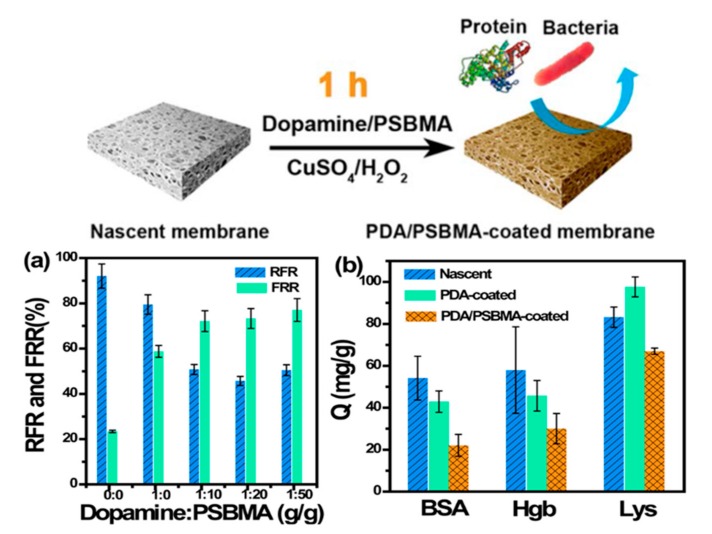
The schematic process of the preparation of a charged mosaic membrane by employing dipole dumbbell- and egg-like microspheres. Reproduced with permission from Reference [175].

Charged mosaic membranes consist of parallel domains of anion-exchange and cation-exchange elements, which allow cationic and anionic substances to transport through the membrane while resisting low molecular weight organics. It is an effective, energy saving, and clean process for separating organics from salt solutions [24,170]. Previously, charged mosaic membranes were prepared with block copolymers, where the micro-phase separation regions were formed for positive and negative charges [171,172]. Ishizu et al. [173,174] prepared charged mosaic membranes with strong acid/strong base or weak acid/strong base microdomains on a microporous substrate membrane by means of an epitaxial process of phase growth. The acid cylindrical microdomains seemed to pass uninterruptedly and vertically through the membrane. With introduction of the charge and domain fixing of ion exchange regions, such mosaic microdomains could selectively transport organic and inorganic solutes through the membranes. For example, the KCl permeability was about 20-fold compared to those of glucose and sucrose, thus this charged mosaic membrane can be used for sugar purification and salts reclaimation. Ni et al. [175] proposed using polymer microspheres to construct charged mosaic membranes, as schematically shown in Figure 22. In this method, the dipole-like microspheres were first prepared and then oriented before the formation of a membrane. The orientation of microspheres favors uninterrupted phase growths perpendicularly through the membrane, and the negatively charged domains and positively charged domains alternately appear both inside and on the surface of the resultant membrane. Moreover, the microspheres can be conveniently modified through cross-linking between and inside the microspheres to enhance the mechanical properties. Liu et al. [54] fabricated a kind of hybrid charged mosaic membranes by a modified metal alkoxide and zwitterionic process. The hybrid precursor was prepared with the reaction of 3-glycidoxypropyltrimethoxysilane (GPTMS) and y-butyrolactone, Ti(O-nBu)_4_, wherein the –BL opened the lactone ring and reacted with the –NH– groups, causing the generation of zwitterionic ion pairs to graft onto the main chain of the polymers. However, the preparation process is usually complex, and the materials are expensive, so it is necessary to develop a convenient method for the preparation of charged mosaic membranes with high performance and low cost.

Zhang et al. [176,177] first proposed the preparation of low-cost charged mosaic membrane by using the interfacial polymerization method. They chose DIA [C_6_H_3_(NH_2_)_2_SO_3_H], polyethylemine (PEI), and tri-mesoyl chloride (TMC) as active monomers; 4-(chloromethyl) benzoyl chloride [CH_2_ClC_6_H_4_(COCl)] and trimethyl amine were used as chemical modification agents; and the interfacial polymerization was conducted at the interface of n-dodecane and the water solution on a porous support layer. The resulted charged mosaic membrane could be used to separate salts and low molecular weight organic molecules. Later, they prepared composite charged mosaic membranes with polyamine [poly(epichlorohydrin amine)] and trimesoyl chloride (TMC) on the polyethersulfone (PES) support membrane by interfacial polymerization [178]. Polyamine with multi-hydroxyl and multi-NH can react with multifunctional acid chloride TMC to form a cross-linked network structure. The high cationic density of polyamine provides sufficient anion-exchange groups, and COOH groups from the hydrolysis of TMC are the cation-exchange groups in the membrane. The interfacial polymerization conditions, such as monomer concentration, reaction time, pH value of the aqueous phase solution, and post-treatment, have been systematically studied. The optimized membrane (preparation conditions: polyamine concentration 0.4%, pH 8.5, TMC concentration 0.5%, interfacial polymerization 30 s, post-treated at 90 °C for 30 min) had a pure water flux of 14.73 L·m^−2^·h^−1^·MPa^−1^, and the separation factor for NaCl/PEG1000 and MgCl_2_/PEG1000 was 11.89 and 9.96, respectively. Deng et al. [179] used a tri-channel PSF hollow fiber ultrafiltration membrane as the support layer. Interfacial polymerization was carried out with polyethylenimine (PEI), 2,5-diamino-benzenesulfonic acid (DIA), basic fuchsin (BF) (aqueous phase monomer), and trimesoyl chloride (TMC) (organic phase monomer). The obtained membrane, containing both sulfonate groups and quaternary ammonium groups, exhibited a charged mosaic membrane. The rejection of membranes to NaCl, polyethylene glycol, Xylenol orange, and methyl chloride was 12.4%, 90.0%, 96.0%, 88.0%, and 88.2%, respectively. The result showed that retentions could be enhanced with the increase of DIA, PEI, and SDS concentrations and decreased with decreasing FB concentration. Zhang et al. [180] also prepared composite charged mosaic membranes on a micro-porous polyethersulfone hollow fiber membrane by interfacial polymerization. They investigated the separation performance of the membrane to binary/ternary mixtures, and found that the separation factors decrease with increasing salt concentration due some interaction between salt and organics. 

**Figure 23:**
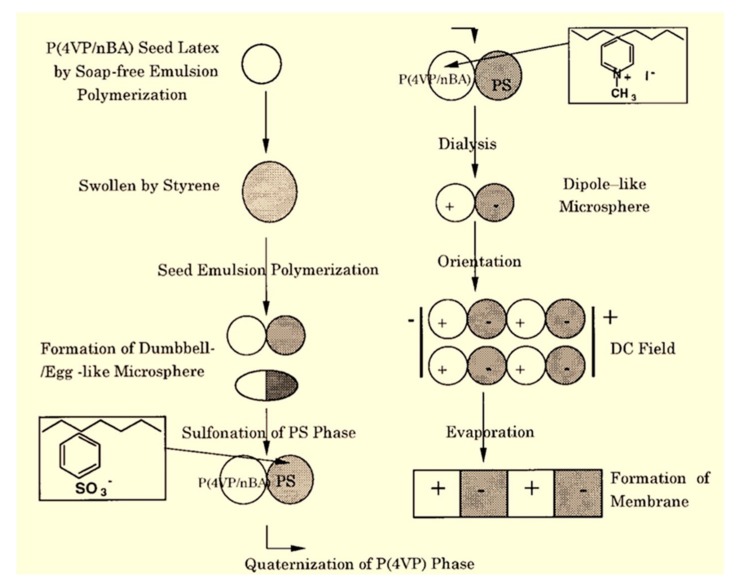
Schematic diagram for the separation mechanism of pristine membranes and charged mosaic membranes. Reproduced with permission from Reference [182].

In order to meet the requirement of large-scale preparation with low cost, the polymer blend and non-solvent induced phase separation method were adopted to fabricate charged mosaic membranes. Higa et al. [53] prepared charged mosaic membranes from laminated structures of charged poly(vinyl alcohol) membranes, where the negatively charged base membranes and positively charged base membranes were alternately stacked. Compared with the Desalton^®^ membrane, there is a much higher permselectivity for salt of this membrane with a relatively lower flux. In addition, the mosaic membranes were fabricated with a 1:1:1 blend of polymers, comprising aminated poly(2,6-dimethyl-1,4-phenylene oxide) (APPO), sulfonated polysulfone (SPSf), and polysulfone (PSf) via a phase inversion process [181]. The pore size of the membrane was tuned with different chemical coagulant baths, and five pure protein components could be separated from the chicken egg white with the mosaic membrane by partitioned free-flow isoelectric focusing (FFIEF). Wang et al. [182] synthesized zwitterion–hydrotalcite (ZHT) by grafting sulfobetaine methacrylate (SBMA) on the surface of Mg/Al hydrotalcite by surface initiated reverse atom transfer radical polymerization (RATRP). In detail, the 3-chloride propyl triethoxy silane (CPS)-modified HT (HT-CPS) (1 g) and SBMA (1.5 mmol) were first mixed in deionized water and ethyl alcohol (10 mL/30 mL) solvent. Then, 2,2′-bipyridine (0.16 mmol), CuCl_2_ (0.32 mmol), and AIBN (0.02 g) dissolved in ethyl alcohol (20 mL) were added to the mixture solution, and the grafting reaction were maintained at 65 °C for 10 h with nitrogen protection. After that, charged mosaic membranes were fabricated with the introduction of ZHT into the polyethersulfone (PES) casting solution via non-solvent induced phase separation (NIPS). This modified membrane had an improved ionic exchange capacity, surface hydrophilicity, and water permeability compared to the original membrane. It also showed high water flux (20.1 L m^−2^ h^−1^ bar^−1^) and excellent dye/salts separation (the retention of Reactive Red 49, MgCl_2_, Na_2_SO_4_, and NaCl was 86.7%, 9.3%, 7.6%, and 0.53%, respectively). The incorporation of zwitterion–hydrotalcite endows the charged mosaic membranes with high charge capacity and nearly neutral charge, reducing the Donnan effect and rendering both cations and anions easily absorbed and transported through the membrane matrix (Figure 23). This result shows that charged mosaic membranes have potential application in dyes purification and salts reclaimation.

**Figure 24:**
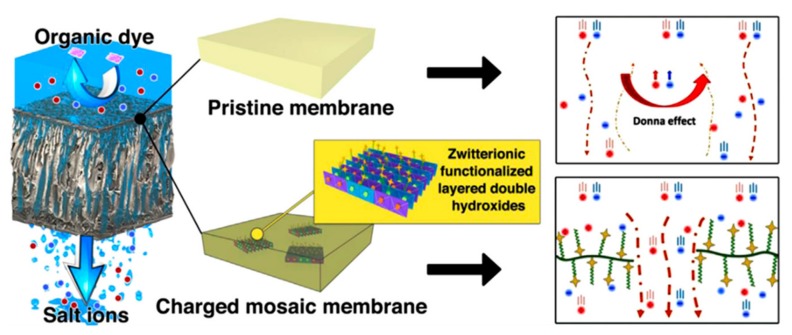
(**Left**) Schematic of the mixed mosaic membranes (MMMs) prepared using LBL assembly. Cationic (blue) and anionic (red) nanotubes are aligned vertically on a support membrane, and a sealing layer is applied to prevent convective flow through the interstitial regions between the nanotubes. Dissolved ions (blue and red spheres) can permeate through nanotubes lined by moieties with the opposite charge, which results in higher ionic permeabilities compared to the permeability of neutral molecules (green spheres). Anionic and cationic polymeric nanotubes were prepared by terminating the multilayer assembly with negatively charged poly(styrenesulfonate) and positively charged polyethylenimine, respectively. (**Right**) Scanning electron micrographs of MMMs. Reproduced with permission from Reference [183].

Recently, some novel methods have been developed for the preparation of charged mosaic membranes with well-defined nanostructure and ion channels, which would provide an effective pathway for the separation of organics/salts mixtures. Rajesh et al. [183] reported an interesting work, in which polymeric nanotubes with a positively/negatively charged outer layer (polyethylenimine/polystyrenesulfonate) were prepared by the layer-by-layer assembly method with track-etched membranes as sacrificial templates. Subsequently, the mixed mosaic membranes were obtained by depositing both types of nanotubes on a porous support membrane. Scanning electron microscopy showed that the nanotubes were vertically aligned without overlap between adjacent elements, and the nanotubes spanned the thickness of the mixed mosaic membranes (Figure 24). The hydraulic permeability of the mixed mosaic membranes is 8.0 L m^−2^ h^−1^ bar^−1^, and ionic solutes transport through the membrane more easily than similarly sized neutral molecules. This result demonstrates that it is a useful method for fabricating charged mosaic membranes with well-defined nano-structures and chemical functionality for salts/organics separations. Recently, a conjugate electrospinning method was employed for the preparation of charged mosaic membranes [184]. The negatively charged sodium polystyrene sulfonate (PNaSS) and positively charged poly(4-vinyl pyridine) were selected as exchange elements, and polyvinyl alcohol was used as the matrix and cross-linked with formaldehyde. The membrane structure was controlled by the electrospinning parameters, such as the concentration of spun solution (the concentration of total polymers was constant at 11 wt %, and the weight ratio of poly(4-vinyl pyridine)/polyvinyl alcohol was selected at 1/9), collecting speed (0.17~0.60 m/s), rate of solution supply (1~5 mL/h), and the distance between the two nozzles (25~40 cm). It was found that the alignment of composite nanofibers was mainly dominated by the concentration of the polyelectrolyte. The alignment degree decreased with increasing PNaSS concentration. Since inkjet printing technology can rapidly and precisely deposit functional materials onto a substrate surface, this has becoming a promising method for the preparation of membranes [185,186]. Gao et al. [187] fabricated charged mosaic membranes by combining the inkjet printing and template synthesis techniques. Composite inks containing polyvinyl alcohol and poly(diallyldimethylammonium chloride)/poly(sodium 4-styrenesulfonate) were used to construct oppositely charged domains on the surface of a polycarbonate track-etched membrane. The resultant membrane possessed an overall neutral charge, which had selective transport for ionic solutes. This method can be extended to a wide range of matrix materials and functional components for preparing mosaic membranes with various patterned surface chemistries and structures.

## 4. Conclusions and Perspectives

Nanofiltration has become one of the main technologies to tackle the problems of water scarcity and energy shortage worldwide. It is highly demanded to further improve the separation performance of membranes and reduce the energy consumption and operation cost in the application process. Inspired by biological cell membranes, materials containing “ion pairs” have been demonstrated to be promising candidates to construct advanced nanofiltration membranes with high permeability, selectivity, and antifouling properties. Multilayer polyelectrolyte membranes prepared by LBL assembly methods have attracted the interest of researchers in the past decade, and have shown promise for the fabrication of perfect thin films in the nanometer scale. Homogeneous polyelectrolyte complex membranes (HPECMs) made from water-dispersible HPECs are thought to be a unique and convenient way to prepare high-performance nanofiltration membranes. The “water channels” might be produced between hydrophilic and charged HPEC nanoparticles, which would facilitate water molecule transport through membranes while resisting the permeation of salts. Zwitterionic materials are recognized as very promising membrane materials because they can generate “free water” hydration shells via the attraction of ion pairs, which have superior hydrophilicity and strong antifouling properties. Charged mosaic membranes are well known as membranes containing oppositely charged groups within the matrix, and the array of ion pairs induces fast electrolytes transport across the membrane, leading to an efficient separation of salts and organic molecules. In this review, we introduced the materials containing “ion pairs” and summarized the approaches to construct their membranes, including LBL deposition, surface coating, interfacial polymerization, surface grafting, and biomimetic adsorption. In order to push the membranes containing “ion pairs” applied in actual nanofiltration processes, it is necessary to simplify the synthesis of materials, reduce the membrane preparation cost, and realize the manufacture of membrane modules for large-scale use. 

Membranes containing “ion pairs” show high separation capability and antifouling property, although hybrid membranes have yet not been studied extensively. By utilizing the unique interaction of ion pairs, species with special functionalities can be incorporated into the membranes, e.g., hydrophilic/hydrophobic neutral polymers, inorganic nanomaterials, and biological activity materials can be used to further enhance the membranes’ permselectivity, thermal-/mechanical stability, bio-compatibility, and fouling resistance.

As discussed in this article, membranes containing “ion pairs” can be prepared with different methods, and have been utilized in water treatment as well as substance separation and purification in organic systems. It is very important to understand the interaction between the solvent, solute, and membranes, and what roles “ion pairs” play in different application conditions. It is highly recommended to develop advanced instruments and simulation techniques to characterize and analyze the variation of membrane microstructures in complex systems, i.e., investigating mixture feed solutions with multiple foulants to imitate actual situations. Establishing the relationship between the membrane materials, structures, and performances is very helpful to understand the transport and antifouling mechanism of nanofiltration membranes containing “ion pairs”. Furthering this understanding would provide more fundamental knowledge for exploiting advanced nanofiltration membranes for water treatment and substance separation applications.

## Figures and Tables

**Table 1:** Summary of materials, method, and performance of polyelectrolyte membranes containing ion pairs.

Membrane Material		Fabrication Method	Solvent	Permeance (L m^−2^ h^−1^ bar^−1^)	Solute	Rejection (%)	Ref.
Material	Substrate						
(PVA/PVS)_60_	PAN/PET supports	LBL	Water	~0.1	NaCl	93.5	[40]
					Na_2_SO_4_	~97.0	
					MgSO_4_	~99.0	
					MgCl_2_	~82.0	
(PSS/PDDA)_4.5_	Porous alumina supports	LBL	Water	18.2	NaCl	40.0	[77]
					glycerol	18.0	
					glucose	64.0	
					sucrose	97.2	
					raffinose	98.9	
(PSS/CS)_4.5_	Porous alumina supports	LBL	Water	13.0	glycerol	4.0	[77]
					glucose	46.0	
					sucrose	89.0	
					raffinose	97.0	
(PSS/PAH)_4_-PSS	Porous alumina supports	LBL	Water	21.7	NaCl	13.0	[78]
				15.6	Na_2_SO_4_	95.0	
				8.7	MgSO_4_	90.0	
				13.0	CaCl_2_	48.0	
(PAA/PAH)_4_PAA	Porous alumina supports	LBL	Water	1.5	NaClNa_2_SO_4_	Cl^−^: 12.9SO_4_^2−^: 93.2	[79]
(PAA/PDDA)_4_PAA				12.2		Cl^−^: −4.4SO_4_^2−^: 24.0	
(PSS/PAH)_4_PSS				20.0		Cl^−^: −8.7SO_4_^2−^: 78.0	
(PSS/PDDA)_4_PSS				20.8		Cl^−^: −15.5SO_4_^2−^: 92.3	
(PDDA/SPEEK)_10_	PAN	LBL	Water	68.8	NaCl	8.95	[84]
				77.1	Na_2_SO	10.5	
				10.8	Na_2_HPO_4_	55.7	
(PSS/PDDA)_4_PSS	Porous alumina supports	LBL	Water	30.4	NaCl NaF	Cl^−^: 9.5F^−^: 73.1	[88]
(PSS/PAH)_4_PSS				40.8		Cl^−^: 16.2F^−^: 21.8	
(PAH/PAA)_4_PAH	PES-UF	LBL	Water	/	Na_2_SO_4_	~24.0	[90]
					NaCl	~61.0	
					sulfamethoxazole	~68.0	
(PAH/PAA 3.5)_10_	PSF-UF	LBL	Water	~0.5	NaCl	~99.0	[91]
(CS/PSS)_3_-(PAH/PSS)_2_	PAN-UF	LBL	Water	~1.2	Glucose	63.0	[92]
					Maltose	99.2	
					Oligosaccharides	100.0	
(PAH/PSS)_1_-(PAH/PSSMA)	PAN-UF	Dynamic LBL	Water	13.6	Na_2_SO_4_	91.6	[93]
				15.3	NaCl	33.1	
				14.5	MgSO_4_	86.4	
				15.3	MgCl_2_	66.2	
(PAH/PAA)_6_	Hollow Fiber Silica with a separation skin layer of PES	LBL	Water	~3.0	Na_2_SO_4_	~46.0	[48]
					NaCl	~9.0	
					CaCl_2_	~27.0	
					Atenolol	~76.0	
					sulfamethoxazole	~62.0	
					naproxen	~72.0	
					atrazine	~64.0	
					bezafibrate	~78.0	
(PSS/PAH)_7_	Porous alumina supports	LBL	Water	~11.3	Glycine	34.5	[94]
					Serine	53.0	
					Alanine	62.4	
					Glutamine	98.7	
(PDDA/SPEEK)_5_	PAN-UF	LBL	IPA	2.9	Rose Bengale	91.0	[37]
				2.0	Acid Fuchsine	98.0	
				12.9	Crystal Violet	17.0	
				12.5	Methyl Orange	50.0	
(PDDA/PAA)_20_	PAN-UF	LBL	DMF	~0.1	Rose Bengale	~92.0	[98]
			ACN	~2.1	Rose Bengale	~87.0	
			IPA	~0.1	Bromothymol Blue	~58.0	
			IPA	~0.2	Acid Fuchsine	~76.0	
(PDDA/PSSH)_10_	PAN-UF	LBL	IPA	~0.1	Rose Bengale	~99.0	[99]
					Acid Fuchsine	~99.0	
					Bromothymol Blue	~89.0	
(PDDA/PVSH)_5_	PAN-UF	LBL	IPA	~1.6	Rose Bengale	~99.0	[99]
				~2.1	Acid Fuchsine	~99.0	
				~1.4	Bromothymol Blue	~84.0	
(PDDA/HPE)_1_	PAN-UF	LBL	IPA	~1.2	Rose Bengale	~98.0	[101]
			Water	~5.4	Rose Bengale	~99.0	
			ACN	~4.3	Rose Bengale	~94.5	
			THF	~1.2	Rose Bengale	~90.0	
QCMC/CMCNa	PSF-UF	Surface coating and chemical cross-linking	Water	~1.4	K_2_SO_4_	87.7	[108]
					Na_2_SO_4_	70.6	
					MgSO_4_	27.6	
					NaCl	22.8	
					CuCl_2_	14.7	
					MgCl_2_	10.8	
					XO	98.7	
PDDA/CMCNa	PSF-UF	Surface coating and chemical cross-linking	Water	~1.9	NaCl	24.2	[108]
					K_2_SO_4_	91.8	
					XO	99.3	
PDMC/CMCNa	PSF-UF	Surface coating and chemical cross-linking	Water	~3.0	NaCl	2.0	[109]
					K_2_SO_4_	97.0	
Sulfated CS/DSS	PSF-UF	Surface coating and chemical cross-linking	Water	~6.4	Methyl Blue	99.9	[115]
					Na_2_SO_4_	93.4	
					K_2_SO_4_	92.2	
					MgSO_4_	41.9	
					NaCl	13.8	
					KCl	13.5	
					MgCl_2_	~10.5	
PCMVImTf2N/P(AA1-*co*-ANx)	-	Surface coating	Water	5.4	NaCl	6	[117]
				4.6	Na_2_SO_4_	59.5	
				6.3	MgCl_2_	0.1	
				16.3	Methyl orange	99.9	
				14.7	Methyl violet	6.5	
PEI/SA	PSF-UF	Surface coating and chemical cross-linking	Water	~2.2	Na_2_SO_4_	6.6	[122]
					MgSO_4_	~25	
					NaCl	37.9	
					MgCl_2_	94	
PEI/SA-MWCNT				~4.5	Na_2_SO_4_	~37.9	
					MgSO_4_	~26	
					NaCl	36.9	
					MgCl_2_	93.5	
PDDA/CMCNa-GO	PVDF	Surface coating and chemical cross-linking	Water	8.2	NaCl	38.6	[123]
				8.9	Na_2_SO_4_	62.1	
				7.0	MgCl_2_	12.2	
				6.9	MgSO_4_	22.0	

**Table 2:** Summary of materials, method, and performance of membranes containing zwitterionic ion pairs.

Membrane Material		Fabrication Method	Solvent	Permeance (L m^−2^ h^−1^ bar^−1^)	Solute	Rejection (%)	Ref.
Material	Substrate						
Sulfobetanine zwitterionic MPDSAH	Polyamide membrane	Conventional free radical graft polymerization	Water	6.5	Na_2_SO_4_	92.0	[71]
Sulfobetanine zwitterionic PSVBP	Polyamide membrane	Conventional free radical graft polymerization	Water	6.5	NaCl	99.7	[127]
Carboxybetaine zwitterionic CBMA	Polyamide membrane	Conventional free radical graft polymerization	Water	5.7	NaCl	98.0	[32]
Phosphobetaine zwitterionic MPC	Polyamide membrane	Atom transfer radical polymerization	Water	3.2	NaCl	90.0	[138]
Iodopropionic/iodomethane	Polyamide membrane	*N*-alkylation reaction and quaternization reaction	Water	~4.6	MgSO_4_	~78.0	[141]
					Na_2_SO_4_	~52.0	
					MgCl_2_	~60.0	
					NaCl	~45.0	
AEPPS-PIP/TMC	PSF-UF	Interfacial polymerization	Water	7.2	K_2_SO_4_	97.0	[33]
					MgSO_4_	90.0	
					NaCl	30.0	
PIP/TMC/AEPPS	PSF-UF	Two-step interfacial polymerization	Water	9.6	Na_2_SO_4_	99.5	[142]
					MgSO_4_	98.5	
					NaCl	43.9	
AEPPS/TMC	PSF-UF	Interfacial polymerization	Water	8.4	K_2_SO_4_	97.1	[144]
					erythromycin	96.5	
					NaCl	<20.0	
PEI-g-SBMA/TMC	PES-UF	Interfacial polymerization	Water	13.2	Na_2_SO_4_	~50.0	[145]
					MgSO_4_	~48.0	
					MgCl_2_	~47.0	
					NaCl	~12.0	
					Methylene blue	~85.0	
					Orange GII	~90.0	
					Congo red	~96.0	
					Methyl blue	~100.0	
Zwitterionic polyelectrolyte nanoparticles (ZPNPs)-PIP/TMC	PSF-UF	Interfacial polymerization	Water	~10.0	Na_2_SO_4_	~96.5	[149]
					NaCl	~10.0	
AEPPS-MPD/TMC	PSF-UF	Interfacial polymerization	Water	3.6	NaCl	98.3	[34]
Zwitterionic colloid nanoparticles (ZCPs)-MPD/TMC	PSF-UF	Interfacial polymerization	Water	~2.5	NaCl	96.5	[151]
Zwitterion-CNT/MPD/TMC	PES-UF	Deposition and interfacial polymerization	Water	~1.3	NaCl	98.6	[152]
GO-PSBMA/polyethersulfone (PES)	-	Phase inversion	Water	~12.0	Na_2_SO_4_	~10.0	[154]
					MgSO_4_	~9.0	
					MgCl_2_	~7.6	
					NaCl	~4.0	
					RR49	~97.0	
					RB5	99.2	
Sulphobetaine tri-copolymer (PDHD)	PSF-UF	Surface coating and chemical cross-linking	Water	~8.0	MgCl_2_	96.5	[155]
					NaCl	48.5	
Zwitterionic colloid particles (ZCPs)	PSF-UF	Surface coating and chemical cross-linking	Water	~4.0	PEG1000	~96.0	[150]
					PEG600	~88.0	
					PEG200	~60.0	
					Na_2_SO_4_	~20.0	
Carboxybetaine bi-copolymer (PCH)	PSF-UF	Surface coating and chemical cross-linking	Water	~8.0	Na_2_SO_4_	91.8	[47]
					MgSO_4_	33.3	
					MgCl_2_	23.5	
					NaCl	3.6	
					PEG800	93.4	
PDDA/PSS, PSBMA	Sulfonated PES hollow fiber silica	LBL	Water	4.5	Na_2_SO_4_	~98.0	[158]
					NaCl	~40.0	
					CaCl_2_	~20.0	
Zwitterionic amphiphilic copolymers PTFEMA-r-SBMA	PVDF 400R UF	Self-assembly	Water	8.4	Methyl Orange	~30	[163]
					Brilliant Blue R	~100	
